# *N*^6^-methyladenosine (m^6^A) dysregulation contributes to network excitability in temporal lobe epilepsy

**DOI:** 10.1172/jci.insight.188612

**Published:** 2025-07-22

**Authors:** Justine Mathoux, Marc-Michel Wilson, Sujithra Srinivas, Gabrielle Litovskich, Leticia Villalba Benito, Cindy Tran, Jaideep Kesavan, Aileen Harnett, Theresa Auer, Amaya Sanz-Rodriguez, Mohammad Kh. A.E. Alkhayyat, Mairéad Sullivan, Zining Liu, Yifan Huang, Austin Lacey, Norman Delanty, Jane Cryan, Francesca M. Brett, Michael A. Farrell, Donncha F. O’Brien, Pablo M. Casillas-Espinosa, Eva M. Jimenez-Mateos, Jeffrey C. Glennon, Mary Canavan, David C. Henshall, Gary P. Brennan

**Affiliations:** 1 Department of Physiology and Medical Physics, RCSI University of Medicine and Health Sciences, Dublin, Ireland.; 2 FutureNeuro Research Ireland Centre for Translational Brain Science, and; 3 UCD School of Biomolecular and Biomedical Science, University College Dublin, Belfield, Dublin 4, Ireland.; 4 UCD Conway Institute, University College Dublin, Dublin, Ireland.; 5 Department of Neuroscience, Central Clinical School, Monash University, Melbourne, Victoria, Australia.; 6 UCD School of Medicine, University College Dublin, Belfield, Dublin, Ireland.; 7 School of Pharmacy and Biomolecular Sciences, RCSI University of Medicine and Health Sciences, Dublin, Ireland.; 8 Department of Neurology,; 9 Department of Neuropathology, and; 10 Department of Neurosurgery, Beaumont Hospital Dublin, Dublin, Ireland.; 11 Department of Neurology, The Alfred Hospital, Commercial Road, Melbourne, Victoria, Australia.; 12 Discipline of Physiology, School of Medicine, Trinity College Dublin, The University of Dublin, Dublin, Ireland.; 13 Translational Immunopathology, School of Biochemistry and Immunology and School of Medicine, Trinity College Dublin, Dublin, Ireland.

**Keywords:** Cell biology, Neuroscience, Epigenetics, Epilepsy, Mouse models

## Abstract

Analogous to DNA methylation and protein phosphorylation, it is now well understood that RNA is also subject to extensive processing and modification. *N*^6^-methyladenosine (m^6^A) is the most abundant internal RNA modification and regulates RNA fate in several ways, including stability and translational efficiency. The role of m^6^A in both experimental and human epilepsy remains unknown. Here, we used transcriptome-wide m^6^A arrays to obtain a detailed analysis of the hippocampal m^6^A-ome from both mouse and human epilepsy samples. We combined this with human proteomic analyses and show that epileptic tissue displays disrupted metabolic and autophagic pathways that may be directly linked to m^6^A processing. Specifically, our results suggest that m^6^A levels inversely correlate with protein pathway activation. Finally, we show that elevated levels of m^6^A decrease seizure susceptibility and severity in mice. Together, our findings indicate that m^6^A represents an additional layer of gene regulation complexity in epilepsy and may contribute to the pathomechanisms that drive the development and maintenance of hyperexcitable brain networks.

## Abstract 2

This study reveals dysregulated m6A patterning in temporal lobe epilepsy, and links altered m6A to changes in protein expression, neuronal structure and seizure susceptibility across models.

## Graphical abstract

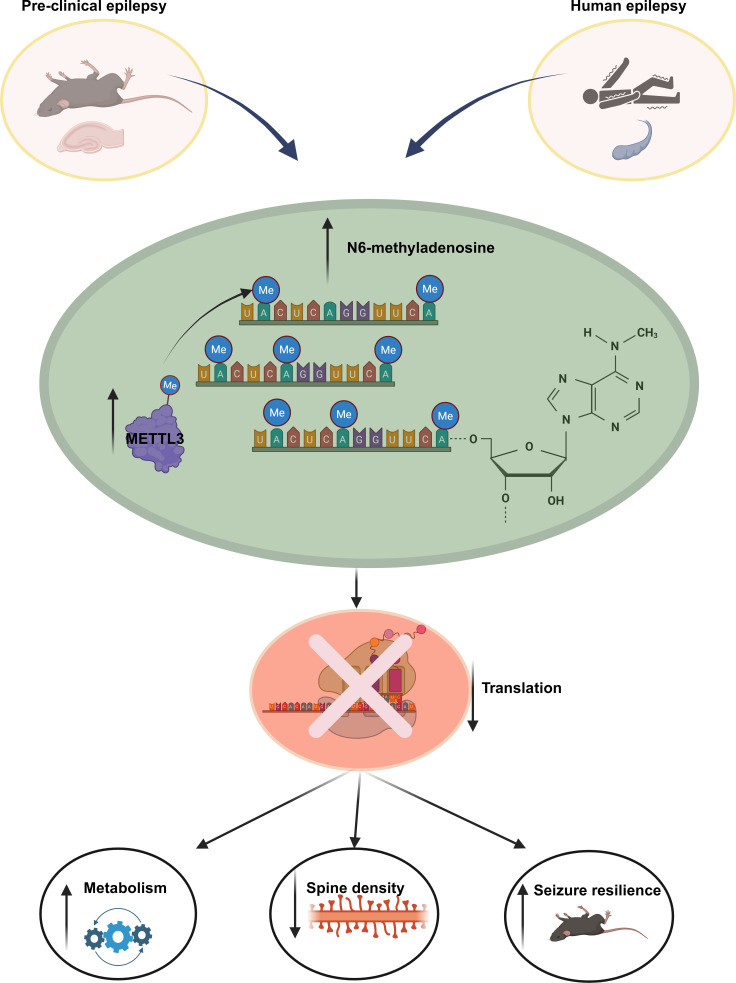

## Introduction

Temporal lobe epilepsy (TLE) is the most common epilepsy syndrome in adults. Often caused by direct brain insults such as traumatic brain injury (TBI) (1), stroke (2–4), central nervous system (CNS) infections, and status epilepticus (SE) (5), it is associated with high rates of drug refractoriness and comorbid symptoms such as persistent learning and memory impairment, depression, and anxiety (6). Injurious brain insults trigger widespread and persistent changes in gene expression and gene expression regulation that govern cellular and network activity, representing an important mechanism of hyperexcitable network formation and maintenance. Indeed, to date, researchers have uncovered extensive dysregulation of the epigenome (7), the transcriptome (8, 9), the miRnome (10), and the translatome (11) in epilepsy. Identifying how gene readout and regulation are affected in epilepsy is critical, not only to improve our understanding of the molecular mechanisms that give rise to the disease but also to identify novel druggable targets to develop mechanism-based therapies that impact the underlying causes and progression of the disease. It is likely that additional, unexplored layers of gene regulation are also affected by epilepsy-provoking insults and contribute to the pathogenic gene expression changes that characterize and drive TLE development.

RNA is subject to substantial covalent modifications, many of which are highly abundant in brain tissues (12–15). These modifications increase functional diversity and the information-carrying capacity of RNA moieties (16). Methylation of adenosine residues at the *N*^6^ position (*N*^6^-methyladenosine, m^6^A) is the most abundant internal modification of messenger RNA (mRNA) (17, 18) and has been reported to regulate RNA subcellular localization (19), RNA stability (20), and translational efficiency (21, 22). Originally described decades ago as a structural modification on ribosomal RNA (rRNA) (23), it has recently gained much attention following technological advancements that allowed transcriptome-wide profiling of m^6^A in cells and tissues. Detection of m^6^A on mRNA and characterization of enzymes that regulate both the catalytic deposition (writers: METTL3, METTL14, WTAP) and removal (erasers: FTO, ALKBH5) of m^6^A uncovered a dynamic modification influenced by cellular needs and stimulus (24). A large group of proteins have now also been identified that recognize and process m^6^A-tagged RNA (readers: YTHDF1–3, ELAVL1, FXRP, and others) (25–27). m^6^A is well conserved and abundant in brain tissue from both humans and mice (17). Although little is known about m^6^A in epilepsy, it has been extensively linked with brain function, having been shown to regulate neuronal development (28), learning and memory (15, 29), and the stress response (30). Given its importance for normal brain function, it is therefore unsurprising that m^6^A regulation is seemingly altered in many neurological conditions, including Alzheimer disease (31, 32), Parkinson disease (33, 34), and TBI (35).

Here, we performed what we believe to be the first comprehensive profiling of m^6^A in epilepsy, using both a preclinical mouse model of the disease — intra-amygdala kainic acid–induced (IAKA-induced) TLE, and hippocampal tissue samples from individuals with drug-refractory TLE. We identify pathways that display common m^6^A dysregulation in both mouse and human, suggesting conserved mechanisms in epilepsy. In addition, we explored the functional significance of m^6^A dysregulation by performing proteomic analyses using the same human tissue and then performing functional assays in vitro using METTL3-overexpressing neurons. Finally, we established a clear link between m^6^A and network excitability by pharmacologically elevating m^6^A levels and assessing seizure resilience in mice. Together, our data suggest that m^6^A is profoundly altered in TLE and directly changes network excitability, potentially via the regulation of metabolic and autophagic pathways.

## Results

### Dysregulation of m^6^A-regulating enzymes in epilepsy.

**Figure 1:**
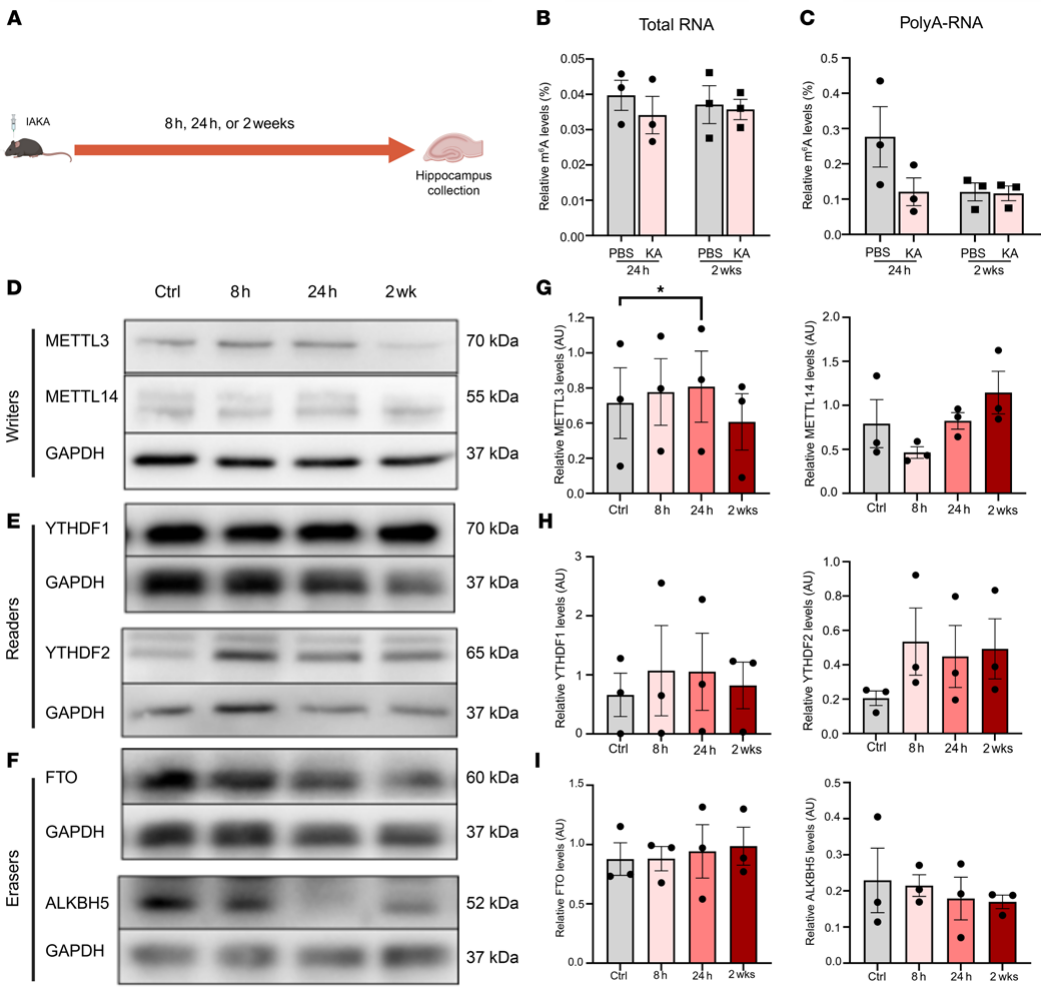
Subtle and transient changes in methyltransferase expression during experimental epileptogenesis. (**A**) Schematic depicting the experimental approach. (**B**) Colorimetric quantification of m^6^A from total RNA isolated from mouse hippocampus at acute (24 hours) and chronic time points after SE (2 weeks) compared to time-matched PBS controls (*n* = 3/group). Two-way ANOVA: f = 0.27, df = (3, 8), *P* = 0.27. (**C**) Colorimetric quantification of m^6^A on polyA-tailed RNA isolated from acute and chronic hippocampal tissue from mice as well as time-matched PBS controls (*n* = 3/group). Two-way ANOVA: f = 2.49, df = (3, 8), *P* = 0.134. (**D**–**F**) Representative Western blot analysis of m^6^A writers (**D**), readers (**E**) and erasers (**F**) at indicated time points (acute: 8 hours, 24 hours, chronic: 2 weeks) (*n* = 3/group). (**G**–**I**) Densitometric quantification of Western blot data for each protein. Pairwise comparison of each time point to the control. METTL3: 8 hours vs. Ctrl *t* = 5.24, Bonferroni-adj. *P* = 0.104; 24 hours vs. Ctrl *t* = 10.14, Bonferroni-adj. *P* = 0.029; 2 weeks vs. Ctrl *t* = –1.51, Bonferroni-adj. *P* = 0.810. No other significant comparisons. *P < 0.05.

We first examined the expression patterns of m^6^A-associated enzymes to gain broad insight into how epilepsy may alter the regulation of this pathway (Figure 1A). Initial quantification of m^6^A levels in total (Figure 1B) and polyA-RNA (Figure 1C) from the hippocampi of acute post-SE (24 hours after KA) and chronic mice (2 weeks after KA) revealed no detectable changes in gross m^6^A abundance at either disease stage. Using quantitative PCR (qPCR) and Western blotting, we next probed the expression of m^6^A writers (METTL3 and METTL14) (Supplemental Figure 1A and Figure 1, D and G; supplemental material available online with this article; https://doi.org/10.1172/jci.insight.188612DS1), readers (YTHDF1 and YTHDF2) (Supplemental Figure 1B and Figure 1, E and H), and erasers (FTO and ALKBH5) (Supplemental Figure 1C and Figure 1, F and I) in acute or chronic mice versus time-matched controls. The abundance of most m^6^A-associated enzymes was relatively stable across both stages of the disease in mice. However, a small but significant increase in METTL3 protein levels was detected in the early acute post-SE time point (24 hours after SE) (Figure 1, D and G). We did not see any differences in mRNA levels for any m^6^A writer, reader, or eraser at any stage of the disease (Supplemental Figure 1, A–C). A second KA model in rats confirmed the findings in mice (Supplemental Figure 1D).

**Figure 2:**
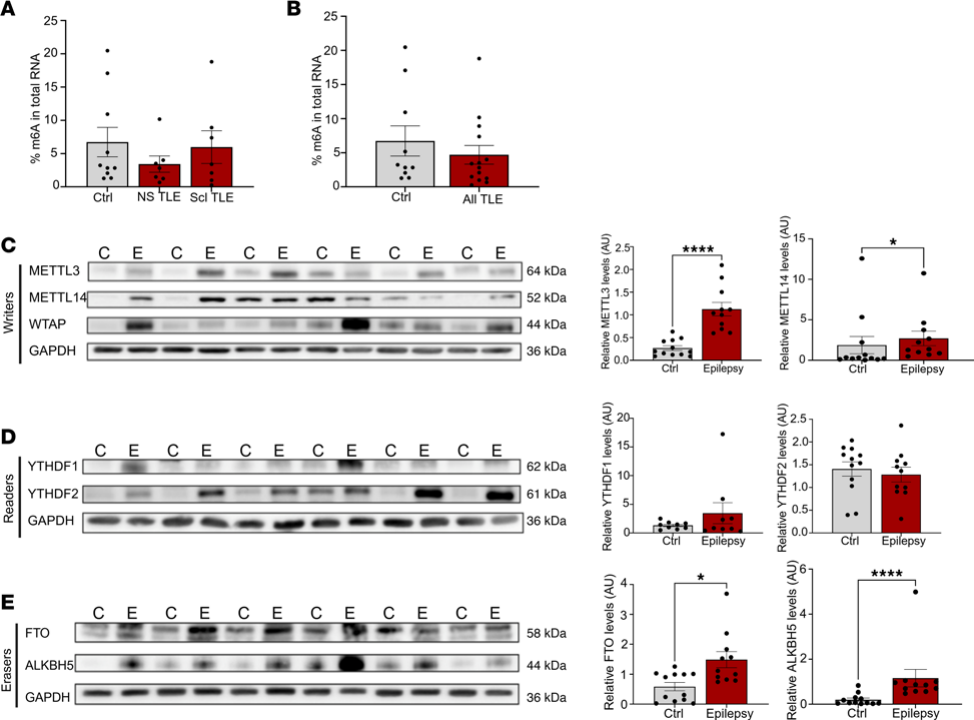
m^6^A regulators are disrupted in resected human TLE tissue compared with controls. (**A**) Colorimetric quantification of m^6^A from total RNA isolated from hippocampal tissue from controls (*n* = 10), nonsclerotic TLE samples (NS-TLE, *n* = 7) and TLE samples with sclerosis (Scl-TLE, *n* = 7). One-way ANOVA, *F* = 0.65, *P* = 0.5321. (**B**) m^6^A quantification from control and combined epilepsy samples. Unpaired 2-tailed Student’s *t* test: *t* = 0.8223; df = 22; *P* = 0.4197. (**C**) Western blot analysis of m^6^A writer proteins METTL3, METTL14, and WTAP in human TLE tissue and non-epileptic controls as well as densitometric quantification of blots (*n* = 11–12/group, epilepsy samples combination of Scl and NS). METTL3: Shapiro-Wilk: Ctrl: *W* = 0.873, *P* = 0.072; Epilepsy: *W* = 0.899, *P* = 0.179; *F* test *P* = 0.046; Welch’s *t* test: *P* = 0.0001, *t* = –5.43. METTL14: Shapiro-Wilk Ctrl: *P* = 0.00005; Epilepsy: *P* = 0.00089; Mann-Whitney test: *U* = 28; *P* = 0.0188. (**D**) Western blot and densitometric quantification of m^6^A readers YTHDF1 and 2 from epilepsy samples and matched controls (*n* = 11–12/group). YTHDF1: Shapiro-Wilk test: Ctrl *P* = 0.817; Epilepsy *P* = 0.0002. Mann-Whitney test: *U* = 40; *P* = 1.0. YTHDF2: unpaired 2-tailed *t* test; *t* = 0.5412, df = 21, *P* = 0.5941. (**E**) Quantification by Western blot of m^6^A erasers FTO and ALKBH5 from human TLE hippocampal tissue and autopsy matched controls (*n* = 11/12 per group). FTO: Shapiro-Wilk Ctrl: *W* = 0.8, *P* = 0.02; Epilepsy: *W* = 0.8, *P* = 0.01; Mann-Whitney test: *U* = 27, *P* = 0.0156. ALKBH5: Shapiro-Wilk: Ctrl: *W* = 0.743, *P* = 0.0023; Epilepsy: *W* = 0.487, *P* < 0.0001; Mann-Whitney test: *U* = 6, *P* < 0.0001. *P < 0.05, ****P < 0.0001.

We next performed the same analyses using human TLE samples compared to autopsy control tissue. Quantification of m^6^A abundance in total RNA was unchanged in both nonsclerotic (NS-TLE) and sclerotic (Scl-TLE) hippocampal samples from patients with TLE compared to control samples (Figure 2, A and B). We then quantified the expression of the same m^6^A-associated enzymes examined previously in the human TLE samples and autopsy controls. Similarly to mice, we detected no significant changes in mRNA levels of any of the m^6^A-associated enzymes in epilepsy (Supplemental Figure 2, A–C). We did, however, detect significant changes at the protein level. These included a significant increase in METTL3 and METTL14 protein abundance in TLE compared with control tissue (Figure 2C) as well as elevated levels of both FTO and ALKBH5 (Figure 2E) in TLE samples. There were no overt differences in the reader proteins YTHDF1 and YTHDF2 in either group (Figure 2D). Overall, in both mouse and human epilepsy, there are changes in the abundance of enzymes regulating m^6^A (notably METTL3 in both mouse and human and FTO and ALKBH5 in human TLE), although no gross changes in m^6^A were detectable using colorimetric assays.

### Temporal and dynamic m^6^A patterning occurs in the hippocampus following SE.

**Figure 3:**
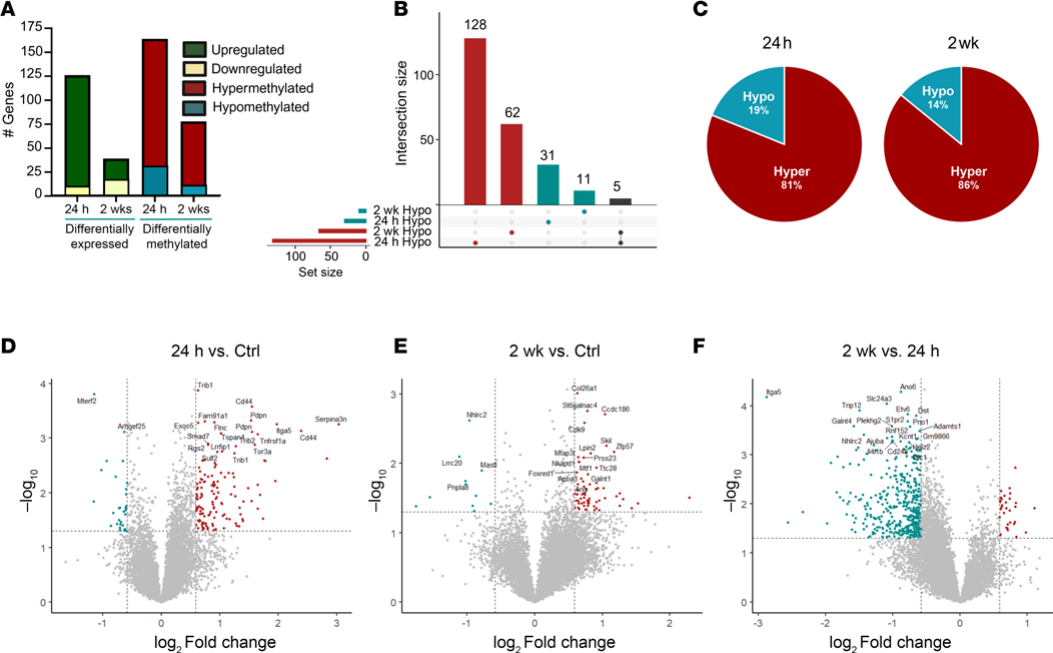
Epilepsy development and progression is associated with altered m^6^A deposition. (**A**) Comparison of the extent of differential gene expression compared to the number of differentially methylated transcripts at acute (24 hours) and chronic (2 weeks) time points in the IAKA model of TLE (*n* = 3–4/group). (**B**) Upset plot demonstrating the dynamic reorganization of the m^6^A epitranscriptome across the various stages of disease development in mice. (**C**) Percentage of differentially methylated transcripts at acute and chronic time points in IAKA mice exhibiting either hyper- or hypomethylation. (**D**–**F**) Volcano plots representations of differentially methylated transcripts from 24-hour (acute) (**D**) and chronic (2 weeks) (**E**) mice compared to controls and (**F**) comparison of differentially methylated transcripts between acute and chronic mouse hippocampus. The top 20 most differentially methylated transcripts are included on each plot.

To examine m^6^A patterning in the hippocampal transcriptome in the IAKA model of TLE, we performed an m^6^A array on RNA isolated from mice at acute (24 hours) and chronic (2 weeks) time points after IAKA as well as time-matched control animals. We first measured gene expression irrespective of m^6^A status by RNA sequencing (RNA-seq) and identified a large number of genes both up- and downregulated at both the acute and chronic time points (Figure 3A, green/yellow bars). As expected, and in line with previous studies, we observed changes in expression patterns of epilepsy-associated genes, including those involved in synaptic transmission, inflammation, and cell death (Supplemental Figure 3, A–D). Profiling of the m^6^A-ome using m^6^A arrays provides transcriptome-wide coverage with single-base resolution. Overall, we identified more extensive m^6^A dysregulation in the acute mice compared with time-matched controls, although dysregulation was also detected in chronic mice (Figure 3A, red/blue bars). There was little overlap in differential m^6^A patterns in acute mice compared to chronic mice, with only 5 hypermethylated transcripts retaining their hypermethylation status into the chronic phase of the disease (Figure 3B). Hypermethylation was more prominent than hypomethylation at both time points (Figure 3C). Overall, in acute mice there were 133 hypermethylated sites and only 31 hypomethylated sites compared with controls, while in chronic mice there were 67 hyper- and 11 hypomethylated sites compared with controls (Figure 3, D and E). Some transcripts contained multiple differentially methylated sites. The majority of differential methylation events were transient, with m^6^A status mostly returning to baseline levels in chronic mice, suggesting potential activity and disease-stage-dependent m^6^A patterning (Figure 3F).

**Figure 4:**
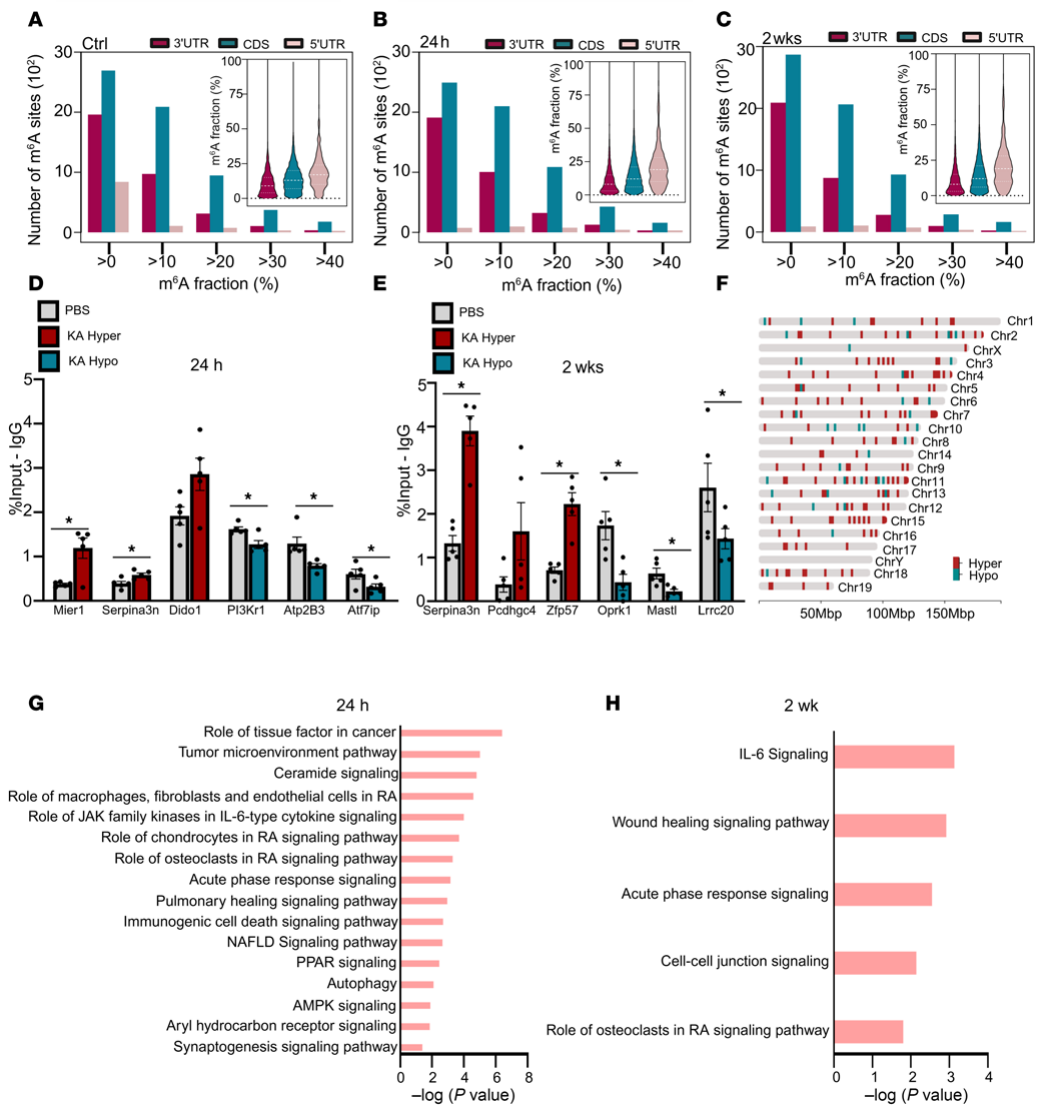
Disrupted m^6^A deposition is associated with epilepsy-associated pathways in mice. (**A**–**C**) Distribution and stoichiometry of m^6^A across transcripts in control (**G**), acute (24 hours) (**H**), and chronic (2 weeks) (**I**) hippocampus across the 3′UTR, coding sequence (CDS), and 5′UTR. (**D** and **E**) meRIP-qPCR validations of selected transcripts identified from m^6^A array to confirm differential methylation in acute (**D**) and chronic mice (**E**) (*n* = 4–5/group). Unpaired 2-tailed *t* test or Mann-Whitney *U* test applied, see Supplemental Table 3 for full statistical summary. *P < 0.05. (**F**) Visual mapping of genomic origin of differentially methylated transcripts from both acute and chronic mice. (**G** and **H**) Ingenuity pathway analysis (IPA) of pathway enrichment associated with differentially methylated transcripts at 24 hours (**G**) and 2 weeks after KA (**H**).

Analysis of m^6^A stoichiometry on mRNA transcripts revealed asymmetric distribution of m^6^A across transcripts, with several-fold higher frequency in the coding sequence (CDS) followed by the 3′UTR than in the 5′UTR in control animals (Figure 4A). Acute (24 hours) and chronic (2 weeks) mice displayed a notable loss of m^6^A in the 5′UTR, with comparable methylation observed in the 3′UTR and CDS at each disease stage (Figure 4, B and C). To validate the array datasets, we performed methylated-RNA immunoprecipitation–qPCR (meRIP-qPCR) on several hyper- and hypomethylated transcripts. We quantified each transcript in the total RNA pool (input sample) and used this to normalize the corresponding m^6^A-modified transcript levels in the immunoprecipitated (IP) fraction, allowing us to assess the accuracy of the array. We observed almost complete concordance of the array data and meRIP-qPCR, which was performed from brain tissue originating from an independent cohort of animals (Figure 4, D and E). Analysis of genomic location of differentially methylated transcripts did not reveal any obvious methylation hotspots, although chromosomes 2 and 11 (Chr2 and -11) displayed more extensive differential methylation patterns (Figure 4F). Some chromosomes displayed only hypermethylation (Chr15), while others showed more extensive hypomethylation (Chr10), suggesting region-specific regulation. Ingenuity Pathway Analysis (IPA) demonstrated that differentially methylated transcripts in acute mice (24 hours) were associated with pathways such as inflammation and inflammation-related cell death and autophagy (Figure 4G), while those in chronic mice (2 weeks) were associated with IL-6 signalling and cellular communication (Figure 4H).

### Human TLE is defined by altered m^6^A patterns regardless of hippocampal sclerosis and sex.

**Figure 5:**
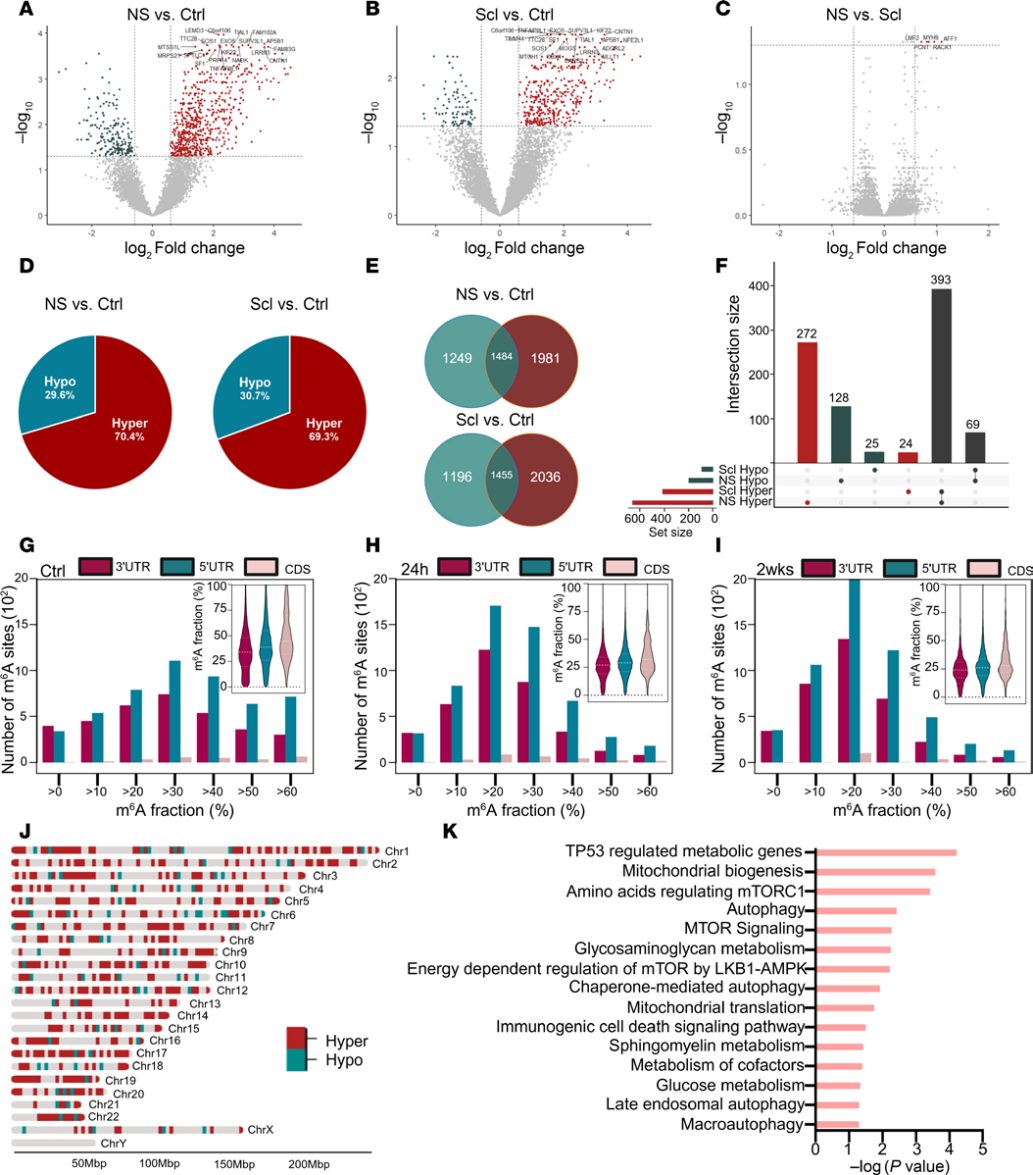
The m^6^A epitranscriptome is extensively disrupted in human TLE. (**A**–**C**) Volcano plot depictions of differentially methylated transcripts in NS human TLE versus controls (**A**), Scl-TLE versus controls (**B**), and NS- versus Scl-TLE (**C**) (*n* = 10 Ctrls, 7 NS-TLE and 7 Scl-TLE). (**D**) Percentages of differentially methylated transcripts in NS- and Scl-TLE that harbor hyper- or hypomethylation patterns compared to controls. (**E**) The overlap of differential methylation between both TLE groups compared to control tissue. (**F**) Upset plot showing overlap of differentially methylated transcripts and those commonly differentially methylated in NS- and Scl-TLE. (**G**–**I**) Distribution and stoichiometry of m^6^A across transcripts in control (**G**), NS-TLE (**H**), and Scl-TLE (**I**) across the 3′UTR, coding sequence (CDS), and 5′UTR. (**J**) Genomic origin of differentially methylated transcripts regardless of detection in Scl or NS tissue samples. (**K**) IPA showing significantly differentially enriched biological processes associated with m^6^A in NS and Scl tissue compared to controls.

Next, we profiled the m^6^A patterning in human TLE using surgically resected hippocampal tissue from individuals with drug-refractory TLE and compared it to age- and sex-matched autopsy control tissue (Supplemental Table 1). m^6^A array profiling revealed significant differential methylation across transcripts in TLE compared with autopsy control. We separated epilepsy samples into those with hippocampal sclerosis (Scl) and those without (NS) based on the neuropathology reports. Both epilepsy groups displayed significantly altered m^6^A patterns compared with controls (Figure 5, A and B) but we found minimal difference in m^6^A patterns between patients with Scl-TLE and those without (NS-TLE) (Figure 5C). Interestingly, m^6^A patterns were not influenced by sex (Supplemental Figure 4A). For both TLE sample types there were over 800 hyper- and more than 300 hypomethylated sites compared with controls. Hypermethylation by abundance was dominant in both the Scl and NS groups compared with control, with roughly 70% of all differential methylation events associated with an increase in m^6^A abundance (Figure 5D). There was significant overlap in the hypermethylated transcripts detected between the NS and Scl groups, suggesting differential m^6^A is a broadly conserved feature of TLE independent of pathology (Figure 5, E and F).

Analysis of m^6^A stoichiometry showed a shift in deposition patterning, with an increase in the number of genes with more than 20% occupancy (Figure 5, G–I). The distribution of m^6^A across transcripts (i.e., 3′UTR, CDS, or 5′UTR) did not change in epilepsy regardless of Scl status (Figure 5, G–I; insets). Genomic location of encoding genes did not reveal any chromosomal hotspots of targeted methylation or demethylation, although Chr17 (which shares evolutionary roots with mouse Chr11) (36) and Chr19 contain regions of genes whose transcripts display enriched m^6^A activity (Figure 5J). IPA of differentially methylated transcripts from the combined epilepsy sample group highlighted autophagic pathways, mTOR signalling, and metabolism as pathways likely to be affected by disrupted methylation patterns in epilepsy (Figure 5K). The current results overall identify extensive m^6^A disruption in human TLE, which potentially affects several epilepsy-associated pathways.

### Commonalities in m^6^A disturbances in experimental and human TLE.

**Figure 6:**
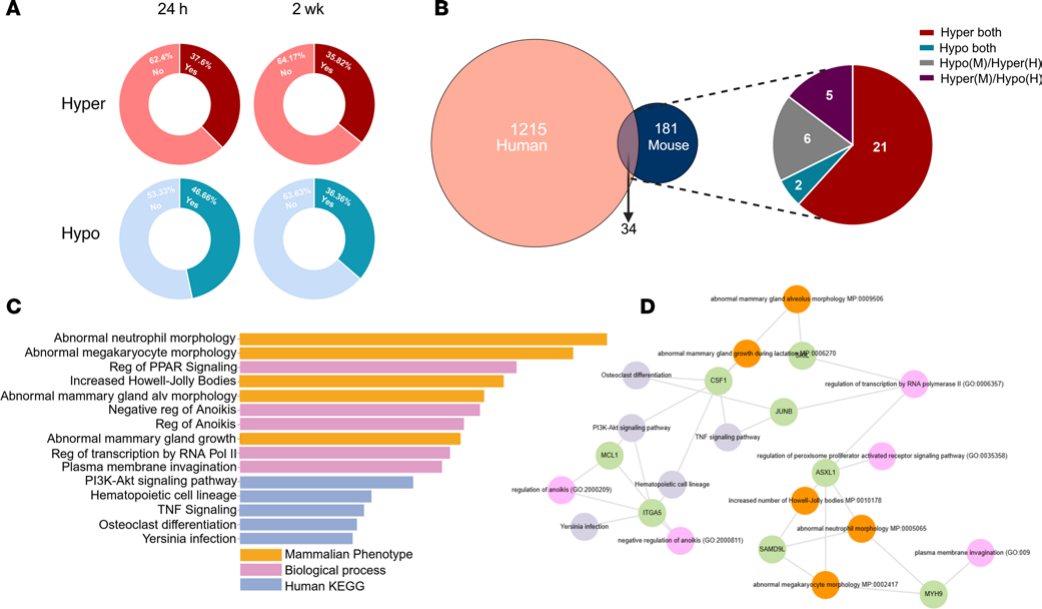
Commonly differentially methylated transcripts in mouse and human are linked to inflammatory and metabolic pathways. (**A**) Donut plot visualization of the presence (Yes)/absence (No) of corresponding human m^6^A orthologous sites in differentially methylated (hyper and hypo) transcripts. (**B**) Overlap of differentially methylated transcripts in mouse and human with directionality and concordance. (**C** and **D**) EnrichR pathway analysis of commonly differentially methylated transcripts from mouse and human TLE.

We next determined whether m^6^A dysregulation seen in experimental TLE is similar to that seen in the human condition by comparing our experimental TLE and human TLE datasets. Our initial analysis investigated whether m^6^A sites profiled in mice possess human orthologs. Between 35% and 45% of the differentially methylated m^6^A sites in experimental mice are orthologous in humans (Figure 6A); however, of those, only a handful were also differentially methylated in human TLE (34 gene transcripts) (Figure 6B). While specific sites were not commonly differentially methylated in both mouse and human epilepsy, there was some overlap in the functionality of transcripts that displayed differential methylation patterns such as inflammatory signalling and cell death pathways (Figure 6, C and D).

### Metabolic pathways are also perturbed at the proteomic level in human TLE.

**Figure 7:**
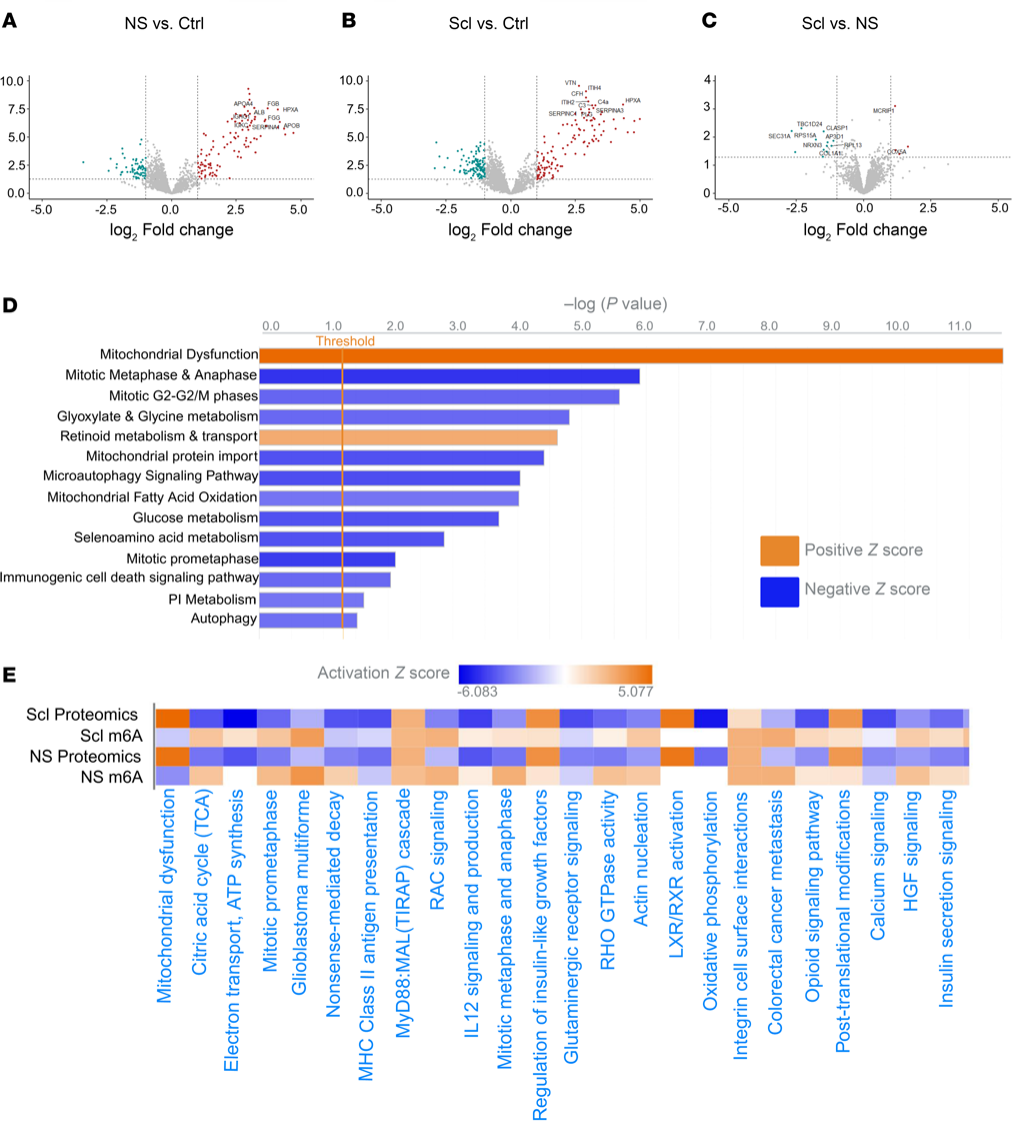
Proteomic analysis of human TLE reveals disruptions in metabolism and autophagic signalling. (**A**–**C**) Volcano plot overview of differentially expressed proteins in NS-TLE (**A**) and Scl-TLE (**B**) compared to controls and NS- versus Scl-TLE (**C**) (*n* = 10 Ctrls, 7 NS-TLE, 7 Scl-TLE). (**D**) IPA of differentially expressed proteins in combined TLE groups and pathway enrichment. (**E**) Representation of directional association between differential methylation and subsequent protein translation.

m^6^A may impact RNA fate in a multitude of ways, including subcellular localization, stability, and/or translational efficiency. To determine whether pathways most likely impacted by differential m^6^A patterning in epilepsy are functionally altered, we performed mass spectrometry (MS) to analyze the human TLE proteome using the same tissue samples used in the m^6^A-profiling experiment. Hierarchical clustering and principal component analysis (PCA) showed good clustering of TLE samples; while control samples also clustered together, it was evident that samples did not separate by sex regardless of condition (Supplemental Figure 4B). A large number of differentially expressed proteins were detected in the TLE groups (NS and Scl) when compared with control tissue (Figure 7, A and B). However, similar to our findings in the m^6^A array, the proteomes of both the NS- and Scl-TLE samples were remarkably similar (Figure 7C). IPA of differentially expressed proteins in the combined epilepsy group (Figure 7D) revealed overall disruption of many metabolism (mitochondrial dysfunction, glucose metabolism) and autophagy-related pathways. We then integrated the human m^6^A data with the human proteomic dataset and found an inverse correlation between m^6^A levels and activation or repression of the corresponding pathways (Figure 7E). These results may suggest that elevation of m^6^A may be a molecular brake to silence pathways.

### Bidirectional modulation of m^6^A alters neuronal metabolism, structure, and seizure susceptibility.

**Figure 8:**
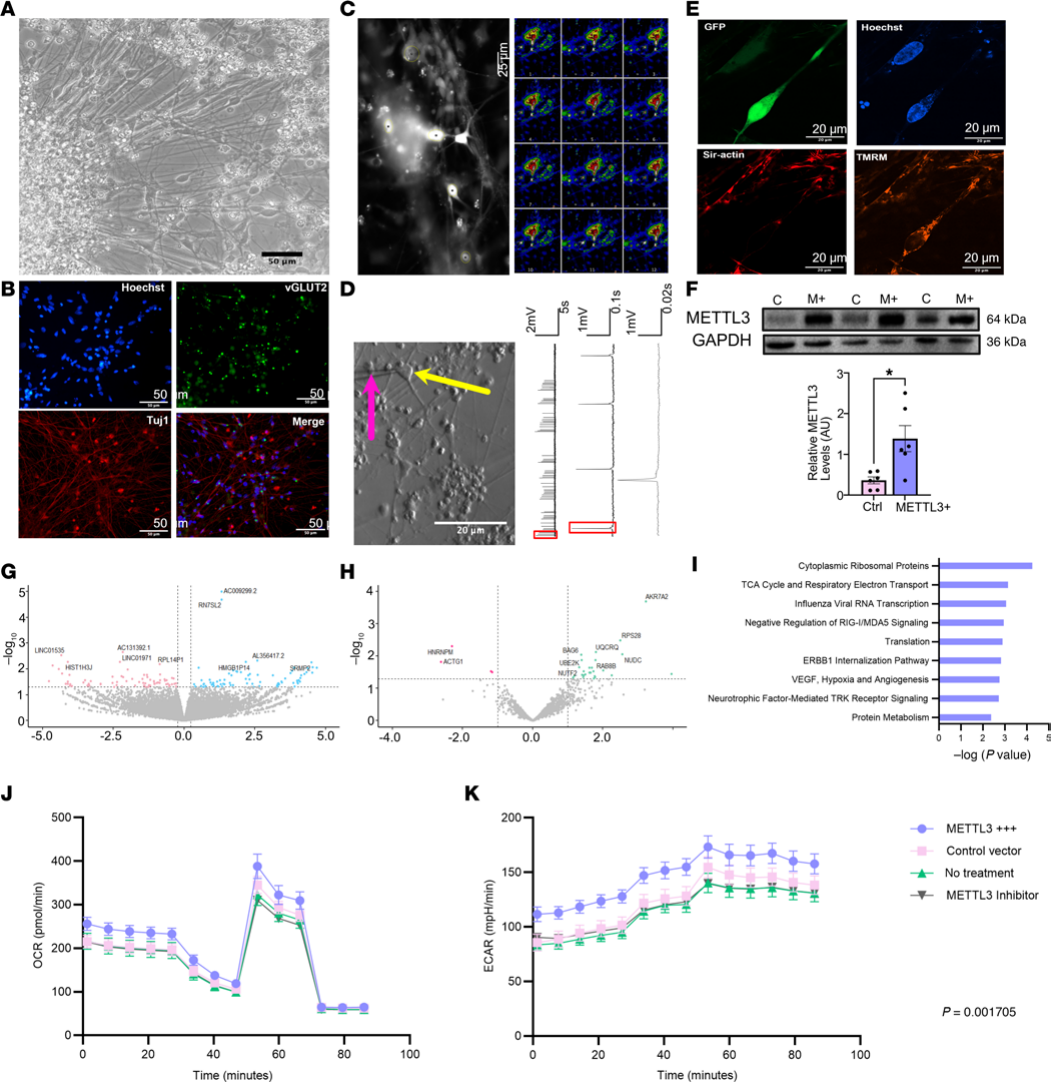
m^6^A regulates hIPSC-derived neuronal metabolism. (**A**) Representative bright-field image of 30-day postdifferentiation mixed hIPSC-derived cortical neurons from healthy control donor. Original magnification, ×400. (**B**) Expression of mature neuronal markers (vGLUT2 and TUJ1) costained with the neuronal marker Hoechst (blue) following 30 days of maturation. (**C** and **D**) Representative mature neuronal activity and firing patterns confirmed by calcium imaging (**C**) and patch-clamp electrophysiology (**D**). (**E** and **F**) AAV transduction of mature neurons was confirmed by imaging for GFP (**E**) and probing METTL3 overexpression by Western blot analysis and densitometric quantification (**F**). Scale bars: 50 μm (**A** and **B**), 50 μm (**C**), and 20 μm (**D** and **E**). METTL3: Mann-Whitney test: *U* = 3, *P* = 0.015 (*n* = 6/group. (**G** and **H**) Volcano plot depiction of differential gene expression (RNA-seq) (**G**), and protein levels (**H**) from neurons overexpressing METTL3 compared to control transduced neurons. (**I**) Gene ontology of differentially expressed genes and proteins by biological process. (**J**) Oxygen consumption rate of neurons overexpressing METTL3 compared to those that do not and those in which METTL3 is specifically inhibited. Comparison of means of normalized group data by 1-way ANOVA followed by Tukey’s multiple comparison testing: *F* = 0.414, *P* = 0.7436, *n* = 3 independent experiments/plates 8 wells/treatment group per plate. (**K**) Extracellular acidification rate of the same neurons. Comparisons of means of normalized group data by 1-way ANOVA followed by Tukey’s multiple comparison testing: *F* = 5.794, *P* = 0.0017, *n* = 3 independent experiments/plates with 8 wells/ treatment group per plate.

The metabolic phenotype common to both mouse and human TLE prompted us to explore the effect of elevated m^6^A on neuronal function. First, using matured human induced pluripotent stem cell–derived (hIPSC-derived) neurons (Figure 8, A–D), we overexpressed METTL3 by transducing cells with a METTL3-overexpressing adeno-associated virus (AAV) vector (Figure 8, E and F). RNA-seq and MS analyses of lysates from these cells detected only subtle changes in both transcription and protein profiles (Figure 8, G and H). However, the pathways most perturbed by METTL3 overexpression were associated with cellular metabolism (Figure 8I). Interestingly, in METTL3-overexpressing cells many downregulated transcripts were noncoding RNAs and pseudogenes (Supplemental Figure 5). Finally, Seahorse analysis demonstrated that human neurons overexpressing METTL3 had elevated rates of glycolysis compared with nontransduced or empty AAV–transduced neurons and this was corrected by pharmacological inhibition of METTL3 (Figure 8, J and K). Overall, our data demonstrate that overexpression of METTL3 elevates the basal metabolic capabilities of even healthy neurons, making them more glycolytically active.

**Figure 9:**
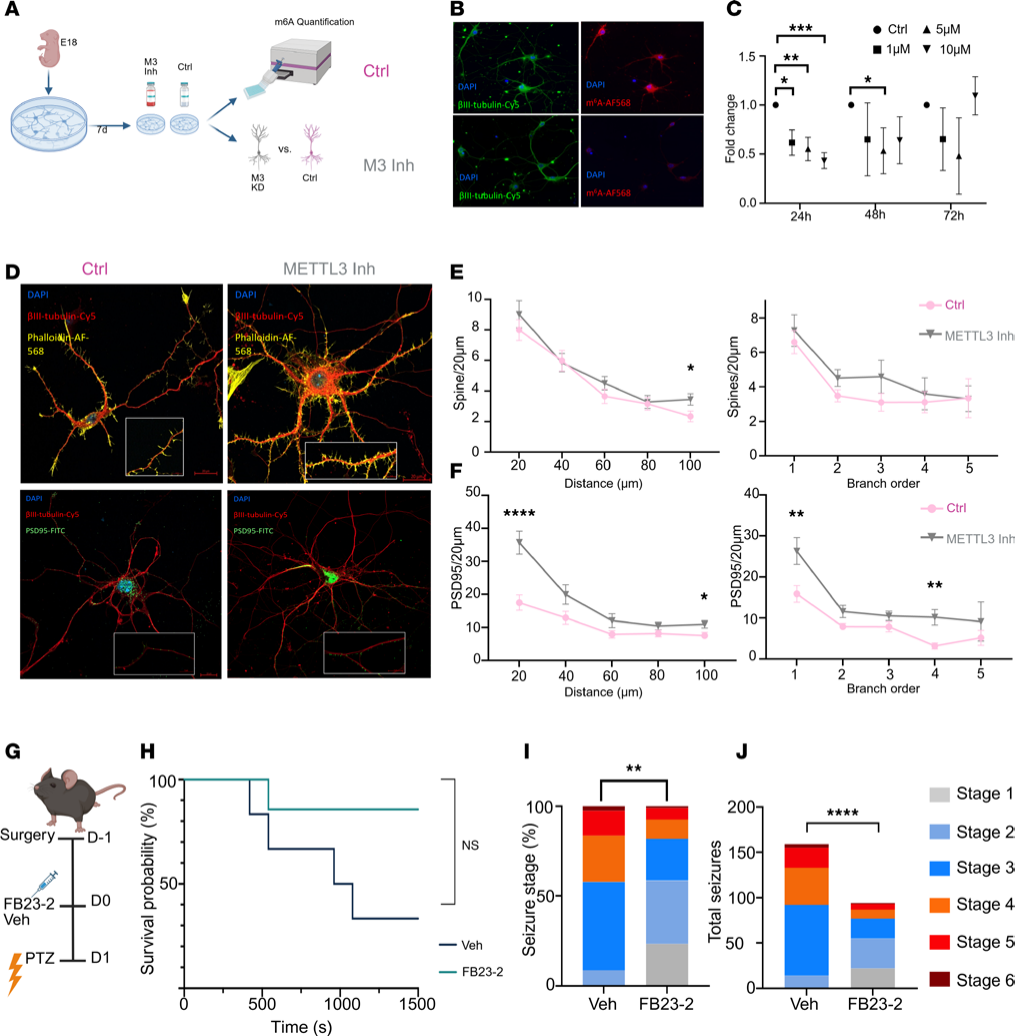
m^6^A regulates neuronal structure and network activity. (**A**) Schematic of experimental approach using primary hippocampal neurons from E18 mouse pups to quantify dendritic spine density in cultures treated with either vehicle or METTL3 inhibitor. (**B**) Representative immunostaining (original magnification, ×10) of neuronal marker BIII_Tubulin and m6A levels following either control or METTL3 inhibitor treatment. (**C**) Colorimetric assay quantification of neuronal m^6^A levels over time following treatment with various concentrations of METTL3 inhibitor. Multiple unpaired 2-tailed *t* tests for each time point, 10 μM 24 hours *t* = 7.049, df = 8, *P* = 0.00032; 48 hours *t* = 1.7, df = 7, *P* = 0.25; 72 hours *t* = 0.55, df = 7, *P* = 0.59. (**D**) Representative confocal ICC images of primary hippocampal neurons stained for βIII-tubulin and with phalloidin to visualize dendritic spines (upper panels) and βIII-tubulin and PSD95 in the bottom panels as a postsynaptic density marker. Inset shows zoomed-in image with puncta staining. Scale bars: 20 μm. (**E**) Dendritic spine quantification characterized by distance from soma binned into 20-μm distances and according to branch order. Distance: 2-way repeated-measures (RM) ANOVA followed by Tukey’s post hoc multiple comparisons 100 μm *t* = 2.174, df = (62, 55), *P* = 0.0335; Branch order: 2-way ANOVA no significance. *n* > 32 dendrites from 22 neurons per group. (**F**) PSD95 puncta quantification according to distance from soma and by branch order. Distance: 2-way ANOVA 20 μm *t* = 4.367, df = (53, 91), *P* = < 0.0001; 100 μm *t* = 2.269, df = (61, 21), *P* = 0.027. Branch order: 2-way ANOVA followed by multiple comparison *t* tests Order 1 *t* = 2.747, df = (63, 00), *P* = 0.03; Order 4 *t* = 3.283, df = (13, 00), *P* = 0.03. *n* > 32 dendrites from 22 neurons per group. (**G**) Experimental design to test whether elevated m^6^A levels alter seizure threshold in mice. (**H**) Kaplan-Meier curve depicting survival rates among vehicle- and FB23-2–treated animals following high-dose PTZ seizure challenge. Kaplan-Meier χ^2^ = 3.4, df = 1, *P* = 0.063. (**I** and **J**) Quantification of the maximum seizure stage reached (**I**) and total seizure numbers (**J**) in vehicle- or FB23-2–treated mice following PTZ administration over a 25-minute recording period, as measured using an adapted Racine scale. Seizure stage/number χ^2^ df = (79.37, 5), *P* < 0.0001. *P < 0.05; ***P < 0.01; ***P < 0.001; ****P < 0.0001.

Next, to assess the effect of m^6^A elevation on neuronal structure we first treated mouse primary hippocampal neurons with a commercially available METTL3 inhibitor (STM2457), and then assessed the effect of elevated m^6^A on dendritic spine density (Figure 9A). METTL3 inhibition led to a detectable increase in m^6^A levels for up to 24 hours in a dose-dependent manner (Figure 9, B and C). We first measured the effect of METTL3 inhibition on spine density as a function of distance from the soma. METTL3 inhibition increased the density of spines, with no clear effect on particular branch order (Figure 9, D and E, and Supplemental Figure 6, A–D). Immunocytochemistry (ICC) for the postsynaptic density protein PSD95 uncovered increased PSD95 immunoreactivity in cultures treated with the METTL3 inhibitor along dendrites (Figure 9, D and F). This increase was apparent in branch orders 1 and 4 (Figure 9F).

We next tested whether recreation of the hypermethylation phenotype seen following SE alters brain excitability. To do this, we treated naive mice with an FTO inhibitor, FB23-2 (37), via intracerebroventricular (ICV) administration prior to a pentylenetetrazol-induced-seizure (PTZ-induced-seizure) challenge (Figure 9G). FB23-2 treatment reduced overall hippocampal FTO levels (Supplemental Figure 7, A and B) and elevated m^6^A levels compared with vehicle-treated animals (Supplemental Figure 7C and Supplemental Figure 8, A–C). A single high dose of PTZ was then administered to both groups of animals and resulting seizure dynamics were quantified using an adapted Racine scale (Supplemental Figure 8F). Overall, we found that FB23-2–treated animals were more likely to survive the PTZ challenge and recording period (Figure 9H). Although FB23-2 had no significant effect on latency to seizure onset (Supplemental Figure 8D), it did reduce the overall severity of seizures experienced by mice compared with vehicle-treated controls (Figure 9I) and the overall number of seizures mice experienced over the recording period (Figure 9J and Supplemental Figure 8E). Together, these in vitro and in vivo data demonstrate a mechanistic link between m^6^A levels and neuronal structure and function.

## Discussion

The present study is, to the best of our knowledge, the first transcriptome-wide and functional characterization of m^6^A in epilepsy. Our study reveals disruption of m^6^A patterning across different disease stages in mouse models of epileptogenesis and chronic epilepsy and significant dysregulation of m^6^A in human TLE. Differentially methylated transcripts in both mouse and human TLE are associated with metabolic, autophagic, and RNA processing pathways. We combined our m^6^A analyses with proteomics in human TLE and discovered that m^6^A patterns are negatively correlated with protein pathway activation or repression. Finally, we demonstrate a clear link between m^6^A abundance and network excitability, with elevated levels of m^6^A increasing the seizure threshold of mice. Taken together, the findings suggest targeting RNA methylation may represent a novel approach to correct gene expression and treat or prevent TLE.

Gene disruption is likely a driver of many of the underlying pathomechanisms that cause the formation and maintenance of hyperexcitable brain networks. Until now, the extent of RNA modification disruption in epilepsy has not been reported; however, several studies have recently identified involvement of m^6^A in the regulation of epilepsy-associated RNA transcripts. These include demonstration that m^6^A regulates NRF2, a transcription factor associated with cellular defence and oxidative stress (38), a result supported by bioinformatic analysis of available sequencing datasets predicting m^6^A-mediated regulation of cell death and metabolism in epilepsy (39). Recently, the m^6^A reader YTHDC2 was also reported to promote the development of TLE via regulation of the astrocytic glutamate-cystine antiporter xCT (40).

Here, our initial characterization of the main enzymes involved in regulation of m^6^A demonstrated that METTL3, the primary catalytic agent of the pathway, was upregulated in both mouse and human tissue. We also detected elevated levels of both FTO and ALKBH5 in human TLE, both of which have been reported to remove m^6^A, suggesting a more complex and nuanced regulation of m^6^A in human epilepsy than in mouse. Additionally, it is conceivable that antiseizure medications may also influence m^6^A patterning, considering some drugs such as valproic acid have been shown to regulate the epigenome. METTL3 and other regulators of m^6^A are dysregulated in a range of other neurological conditions, including stroke, TBI, Huntington disease, and Alzheimer disease, and many studies have shown direct reorganization of the m^6^A epitranscriptome following deliberate or disease-related changes in m^6^A regulator proteins (24, 30, 41–44). Dynamic changes in m^6^A can alter neuronal network properties and communication, thus impacting brain functions like learning and memory and disease-related pathways and possibly seizures (29, 40, 45, 46).

A major finding in the present study was the identification of differentially methylated transcripts and pathways in epilepsy (in both mouse and human). Overall, the number of differentially methylated transcripts in the mouse model were lower than in human TLE. Only 5 gene transcripts were differentially methylated in acute and chronic mice, indicating dynamic and context-dependent methylation events. This may reflect the activation of disease-related pathways during epileptogenesis that may recover in chronic epilepsy when seizures become sporadic. Several studies have found that neuronal activity drives changes in m^6^A activity, so it is likely that the m^6^A profile before and after spontaneous seizure will differ. It is conceivable, therefore, that dynamic m^6^A may contribute to the evolution of the disease over time. m^6^A is usually asymmetrically distributed across mRNAs, with CDS and 3′UTR harboring more m^6^A than the 5′UTR. Interestingly, the stoichiometry of the m^6^A modification in mice revealed extensive loss of m^6^A in the 5′UTR in particular in epilepsy, which may reflect targeted activity of an m^6^A demethylase enzyme or increased processing/turnover of RNA transcripts with 5′UTR m^6^A tags. In the present study, we found no overt differences in the protein levels of YTHDF1 or -2, although it is likely that redundancy exists among readers particularly from the YTH family (27). Dysregulation of various m^6^A readers has been reported in TLE, such as YTHDC2 (40), and in other neurological conditions including genetic variants in the *Fxrp* gene being a direct cause of the neurodevelopmental condition Fragile X syndrome (47). Roughly half of the m^6^A sites profiled in the mouse model have corresponding orthologs in human, making direct comparison of m^6^A patterns between mice and humans difficult. Additionally, we were unable to directly compare identical tissue regions between mouse and human. Despite this, overlapping functions of differentially methylated transcripts were observed, suggesting conserved function of m^6^A in epilepsy across species.

We found that both Scl and NS sample types from the patients with TLE displayed more extensive m^6^A dysregulation compared with controls than was seen in mice. Hypermethylation was prominent and there was large overlap between differentially methylated transcripts in both NS and Scl tissue compared with controls. It is unclear whether this is because m^6^A is not involved in the cell death that defines sclerotic tissue or whether the patterns we observe reflect a bias inherent to profiling surviving tissue. The latter may be more plausible, as several studies have now demonstrated a clear involvement of m^6^A in regulating neurodegenerative pathways and cell cycle events in Alzheimer disease (48, 49) as well as in the pathogenesis of other neurodegenerative diseases like ALS (50), Huntington disease (44), and stroke (51, 52). Furthermore, in our mouse model we found that differentially m^6^A-tagged transcripts were associated with cell death processes in acute mice but not chronically epileptic mice, revealing a stage-specific role for m^6^A in regulating cell death processes. Differential m^6^A tagging was more likely to occur in the CDS in human TLE; however, the overall distribution of m^6^A along transcripts did not change greatly compared to control. IPA of early-stage epileptogenesis in mice implicated disrupted m^6^A deposition on transcripts associated with epileptogenic mechanisms such as cell death and inflammation, while analysis in chronic mouse and human TLE linked differential m^6^A with metabolic processes, autophagy, and RNA processing in line with its role as a homeostatic mechanism. m^6^A has previously been shown to regulate mitochondrial function and other metabolic activities (53) and computational prediction of m^6^A involvement in epilepsy suggested metabolism was intricately linked to m^6^A (39).

Integration of the m^6^A and proteomic datasets interestingly revealed an inverse correlation, i.e., hypomethylation of transcripts resulting in upregulation of protein and vice versa. This supports recent data demonstrating a clear role for m^6^A as a negative regulator of transcript stability (27). The m^6^A array combined with the proteomic analysis made apparent that m^6^A may influence metabolic pathways and processes as well as autophagy and mTOR signalling, which is in line with other reports from other tissues (54). Overexpression of METTL3 in hIPSC-derived neurons did change the expression of metabolism-related transcripts and proteins and increased the rate of glycolysis, supporting previous computational predictions (39). Only subtle changes in the transcriptome, proteome, and metabolism of hIPSC-derived neurons were detected when METTL3 was overexpressed, suggesting m^6^A may couple gene output to translational processes to enable cellular adaptation to environment. This may explain the much greater changes in m^6^A and the resulting proteome seen in epilepsy when cellular activity levels are elevated and disease processes activated.

The current study focusses on the regulatory role of m^6^A modifications and how they may shape the epileptic proteome; however, other posttranscriptional forms of regulation must also be considered. Among these, microRNAs (miRNAs) are well established as key regulators of the proteome and have previously been shown to play a crucial role in epilepsy, including ion channel regulation, metabolism, and autophagy, all of which are implicated in the proteomic changes observed in our study (55, 56). Although miRNAs were not directly profiled here, their potential contributions to the observed proteome must be considered. Further studies are required to elucidate interplay between miRNAs and m^6^A modifications in TLE, as these mechanisms may function synergistically or independently to shape disease-associated proteomic changes.

Finally we found that m^6^A levels are intricately linked with excitability. Reduced m^6^A levels increased dendritic spine density and postsynaptic markers, while elevated m^6^A levels decreased seizure susceptibility and severity in mice. While the precise mechanisms underlying this are still unclear, recent reports have demonstrated a synaptosome-specific m^6^A-ome that may regulate protein translation at the synapse and govern excitability (15). There are certain limitations associated with the current study, including the use of nonadjusted *P* values for the analysis of the mouse m^6^A array. We therefore validated several differentially methylated transcripts using meRIP-qPCR and found good concordance; however, false positives cannot be fully excluded and additional sites are likely being missed. Furthermore, m^6^A changes may be diluted due to the cellular heterogeneity of the input material masking potential cell-specific m^6^A changes. Recent advances may now enable m^6^A profiling at the single-cell level, which could also be explored in epilepsy and other diseases (57). Although efforts are made to compare mouse and human m^6^A data, we used whole mouse hippocampal tissue but only a subsection of resected human tissue due to limitations of availability. The use of behavior as a readout of seizure activity only permits quantification of convulsive seizures and may therefore miss subconvulsive seizure activity. Although we report no differences in m^6^A and proteomic profiles based on sex, the sample size is small and so we are cautious to interpret that sex does not influence m^6^A; instead, we conclude in the context of epilepsy that there is no clear m^6^A signature based on sex. Finally, we have only profiled one RNA modification and it is likely that crosstalk and coordination of diverse RNA modifications exist (16).

Overall, our data suggest that m^6^A represents an additional layer of gene regulation that contributes to the pathomechanisms of epilepsy by regulating RNA stability and protein translation. The elevation in METTL3 in epilepsy may represent an adaptive response, as elevated m^6^A protected mice from seizures. Future preclinical work may now explore whether targeting m^6^A-modifying enzymes may prove beneficial for the treatment of epilepsy. It may also be useful to explore m^6^A dysregulation as a potential novel biomarker for epilepsy and associated comorbidities.

## Methods

### Sex as a biological variable

This study involved both human tissue and animal models. Human hippocampal samples were obtained from individuals with TLE and included both male and female donors. PCA using sex as a variable did not reveal sex-specific clustering or patterns in m^6^A methylation, suggesting minimal influence of sex on the epitranscriptomic landscape in this cohort. All animal experiments were conducted using adult male mice to maintain consistency with previous studies using the IAKA model.

### Mouse models of TLE and acute seizures

#### IAKA in mice.

For all mouse studies, we used 7- to 9-week-old-male C57BL/6JOlaHsd mice, originally from Harlan Laboratories and inbred in house at RCSI. Animals used (matching strains) at University College Dublin (UCD) were sourced from Envigo UK. Prior to IAKA administration, mice underwent a surgical procedure under general anesthesia (isoflurane; 5% induction, 1%–2% maintenance) to implant a surface injection cannula (Bilaney Consultants, custom cut 7.65 mm) over the amygdala (coordinates from Bregma: IA: antero-posterior (AP) = –0.95 mm, lateral (L) = +2.85 mm, ventral (V) = 3.1 mm).

Two to 3 days following this preparatory surgery, mice underwent IAKA microinjection (0.3 μg KA in 0.2 μL PBS; Sigma-Aldrich, K0250) to induce SE. After 40 minutes, mice then received an intraperitoneal (i.p.) injection of lorazepam (Macure, L0227; 8 mg/kg) to reduce seizure severity and reduce mortality. Mice were then allowed to recover in a small animal recovery chamber (Vet-Tech, HE010) at 26°C. At selected time points, animals were euthanized by administering 0.1–0.2 mL 200 mg/mL pentobarbital (Dolethal) via i.p. administration followed by transcardial perfusion with ice-cold 1× RNase-free PBS (Thermo Fisher Scientific, AM9625).

Following IAKA administration, mice generally develop spontaneous seizures after 3 to 5 days, with all animals displaying spontaneous seizures by 2 weeks after SE (58).

#### PTZ in mice.

A subcutaneous dose of PTZ (Sigma-Aldrich, P6500; 80 mg/kg prepared in PBS) was administered 24 hours after 20 μM FB23-2 (VWR International) (37) or vehicle (0.5% DMSO, 40% PEG300, 5% Tween 80, 54.5% H_2_O) treatment. FB23-2 or vehicle was administered via a unilateral ICV administration in 0.4 μL volume. For ICV administration, the stereotaxic coordinates were determined using the Mouse Brain Atlas and verified using methylene blue in preliminary experiments (AP = –0.5 mm, L = –1.1 mm, V = –2.3 mm from Bregma). A 1-mm burr hole was prepared in the exposed skull and a 30-gauge stainless steel cannula connected to a Hamilton syringe was lowered to the correct depth and drug administered. The cannula was held in place for 8 minutes to prevent backflow. The incision was closed with tissue adhesive (Vetbond, IM1469SB). Seizures were recorded for 25 minutes following PTZ administration and scored using an adapted Racine scale (Supplemental Figure 7). Scorers were blinded to treatment. All mice were euthanized at the end of the PTZ protocol.

### Human brain tissue

Hippocampi were collected directly from the surgical theater, frozen immediately in liquid nitrogen, and stored at –80°C. A pathologist confirmed the presence or absence of sclerosis for each tissue sample obtained (59). Hippocampal sclerosis was defined as neuronal loss and gliosis primarily involving the CA1 and CA4/3 subfields with relative resistance of the CA2 region and sparing of the subiculum (if the latter were included in the resected specimen). NeuN immunostaining was employed to determine residual neuronal density and gliosis was determined using GFAP immunoreactivity. Autopsy control samples were sourced from the NIH NeuroBiobank, University of Maryland. Patient and control information is listed in Supplemental Table 1.

### m6A array

RNA was isolated from both mouse and human hippocampal tissue samples as described in the Supplemental Methods. Between 1 and 5 μg of RNA from each sample was sent to Arraystar for single-nucleotide resolution microarray analysis. The array (specific for mouse or human) enables quantification of m^6^A abundance and stoichiometry at approximately 10,000 predefined m^6^A sites across the transcriptome. Briefly, the total RNA was divided into 2 fractions. One fraction was treated with the RNA endoribonuclease MazF to cleave unmodified m^6^A sites, while the other fraction was left untreated. MazF specifically cleaves RNA at unmethylated adenosine residues, allowing for the selective removal of unmodified RNA and preservation of m^6^A-modified sites. The treated and untreated RNA fractions were separately labelled with Cy5 (for MazF-digested RNA) and Cy3 (for the undigested RNA) as complementary RNAs (cRNA) using the Arraystar Super RNA Labelling Kit. These labelled cRNAs were then hybridized onto the Arraystar Human or Mouse m^6^A Single Nucleotide Array (8 × 15K, Arraystar). After hybridization, the arrays were washed and scanned in 2 color channels using an Agilent Scanner (G2505C).

The acquired array images were analyzed using Agilent Feature Extraction software (version 11.01.1). The raw intensity values for the Cy5-labelled (MazF digested) and Cy3-labelled (undigested) cRNAs were normalized using the average log_2_-scaled Spike-in RNA intensities. The Spike-in RNAs, which are synthetic control RNA sequences added at known concentrations, allow for normalization by accounting for technical variations in RNA labelling and hybridization. Log_2_ scaling was used to transform the intensity values into logarithmic scale to enable comparison of fold changes in signal intensities across samples. After Spike-in normalization, we filtered and retained probes with “Present” (P) or “marginal” (M) quality flags (which indicate the reliability of the probe signal compared to background) in at least 7 of the 24 human samples and 6 out of 8 mouse samples for further m^6^A site methylation abundance analysis. Differentially methylated sites between 2 comparison groups were compiled by fold change and statistical significance threshold (*P* < 0.05 [standard for this type of analysis]). For the human array, false discovery rate (FDR) was applied using the Benjamini-Hochberg procedure and adjusted *P* values of less than 0.05 were set as meaningfully significant. Data visualization was performed in RStudio (version 4.3.0 and 4.3.1; https://cran.r-project.org/) (volcano plots [ggplot], chromosome plotting [Chromomap], Upset plots [UpsetR]) and GraphPad Prism (version 9.2.0). Stoichiometric data were plotted using GraphPad Prism. Differentially methylated transcripts were analyzed using Qiagen IPA to identify associated pathways. To analyze the effects of sex on the m^6^A profiles within and between human sample groups, a 3-column (factor) metadata table was created manually by merging a sample ID/condition metadata table, and a sample ID/sex metadata table. PCA was then performed on the processed quantification table and methylation quantification tables using the FactoMineR R library. The PCA plot was then generated using ggplot2, specifically labelling data points indicating condition and sex. ANOVA was then carried out to determine the effect of each factor and a permutational analysis of variance (PermANOVA) using the vegan R library was carried out to determine effects without the assumption of normality.

### meRIP-qPCR

Total RNA was isolated as described in the Supplemental Methods using TRIzol (Invitrogen, 15596026) and quality and concentration assessed by Nanodrop (Thermo Fisher Scientific). Eighteen micrograms of RNA was separated based on Nanodrop quantification from each sample and subjected to fragmentation using an RNA fragmentation kit (Ambion, AM8740) according to the manufacturer’s instructions. This produced RNA fragments between 100 and 200 nucleotides in length (unfragmented RNA, i.e., leftover RNA not used for fragmentation, was kept as input comparison). The fragmented RNA was then incubated overnight at 4°C in IP buffer (10 mM Tris-HCl pH 7.4; Thermo Fisher Scientific, J62848.AK), 150 mM NaCl (Sigma-Aldrich, 71386), and 0.1% IGEPAL CA-630 (Sigma-Aldrich, l8896) containing either anti-m^6^A antibody (Synaptic Systems, 202 003) (17) or IgG (Abcam, ab171870) on a carousel. The antibody–RNA complexes were captured by incubation with washed Protein A/G magnetic Dynabeads (Thermo Fisher Scientific, 10015D) for 2 hours at 4°C with gentle rotation. After several washes to remove loosely bound complexes, m^6^A-tagged RNAs were separated from the beads by elution by competition, which involved incubating the beads with elution buffer (IP buffer, 20 mM m^6^A 5′-monophosphate sodium salt [Sigma-Aldrich, M2780], RNasin [Promega, N2611], nuclease-free water). This was repeated twice and the supernatants pooled and transferred to an RNeasy MiniElute Spin Column (Qiagen, 74204). The samples were then analyzed on a bioanalyzer using the RNA Pico Kit (Agilent, 5067-1511) according to the kit instructions. The purified RNA was then reverse transcribed and qPCR was performed using m^6^A-site-flanking primers (Supplemental Table 2). Input sample was run alongside the IP samples and used as comparison.

### MS-based proteomics

Total protein was extracted as described above and then lysed into smaller peptide fragments using the iST Sample Preparation Kit (PreOmics, P.O.00001) as per the manufacturer’s instructions. Briefly, lysis reagent was added to 1–100 μg of protein from each sample and heated on a dry block incubator (Thermo Fisher Scientific, 15392185) to 95°C with rigorous mixing. Contaminating DNA in the samples was sheared by water bath sonication and samples were then transferred to cartridges and allowed to cool to room temperature (RT). The samples then underwent a digestion step using specific Digest buffer at 37°C for between 1 and 3 hours. The reaction was terminated using a kit-supplied stop solution and samples were then centrifuged at 3,800*g* for 3 minutes and the flow through was collected and discarded. The fraction of the sample bound to the cartridge were then washed twice and eluted with elution buffer twice using centrifugation. The samples were dried completely using a speed vac at 45°C. LC-Load buffer was added to each sample to obtain a 1 g/L concentration. The samples were finally sonicated at RT for 5 minutes and stored at –80°C until needed. Peptide samples were analyzed via liquid chromatography–tandem MS (LC-MS/MS) at the Conway Institute Proteomics core facility, UCD, on a Thermo Fisher Scientific Q-Exactive Mass Spectrometer connected to a Dionex Ultimate 3000 (RSLCnano) chromatography system. Peptides were separated on a C18 home-made column (C18-AQ Dr Maisch Reprosil-Pur, 100 × 0.075 mm × 3 μm) over 60 minutes at a flow rate of 250 nL/min with a linear gradient of increasing acetonitrile from 1% to 27%. The MS was operated in data-independent mode; a high-resolution (70,000) MS scan (300–1600 *m*/*z*) was performed to select the 12 most intense ions and fragmented using high-energy C-trap dissociation for MS/MS analysis.

For proteomic analysis, raw data from the Q-Exactive were processed using MaxQuant (version 2.0.3.0) (60, 61). To identify peptides and proteins, MS/MS spectra were matched against the *Homo* *sapiens* database (https://www.uniprot.org/proteomes/UP000005640; downloaded 2022_01 containing 75,777 entries). Database searches were performed with carbamidomethyl (CAM) as a fixed modification and acetylation (protein N-terminus) and oxidation (M) as variable modifications. Data were then filtered in Perseus (version 1.16.5.0; https://maxquant.net/perseus/). All function calls/commands were carried out using the Perseus interface. Initially a matrix of label-free quantification (LFQ) intensity values were generated for each sample. Filters were then applied to remove values likely to be contaminants, proteins identified in reverse amino acid sequences, and proteins only identified by site. The LFQ values were then log transformed and histograms of the data were generated and checked for normality of data spread. The samples were then grouped by condition (control, Scl-TLE, and NS-TLE). Further filtering excluded proteins that were detected in less than 7 samples per group. Missing values were imputed using the “replace missing values from normal distribution” command. Pearson’s correlation was performed to scrutinize correlation between groups. Preliminary hierarchical clustering was performed to determine clustering and heatmaps were subsequently generated based on this clustering. Further processing and data visualization was carried out in RStudio using the limma R package for differential (protein) expression analysis, which incorporates FDR correction using the Benjamini-Hochberg method, ClusterProfile for enrichment analysis, and ggplot2 for graph generation. The resulting differentially expressed proteins were compared with various functional gene set databases in functional analysis/pathway enrichment analysis using IPA (Qiagen) or EnrichR. Sex differences were analyzed in the same way as described above (see *m^6^A array*).

### ICC

#### hIPSC ICC.

Glass coverslips with cells were transferred to prewarmed DMEM/F-12 (Gibco, A4192101) in a new plate. The cells were then fixed by covering wells with 4% paraformaldehyde (PFA) (Merck, 1004968350) in PBS and left to be incubated for 20 minutes at RT. The coverslips were then washed 3 times with PBS, blocked with 1% BSA (Merck, A9647) in PBS, and then permeabilized with 0.2% Triton X-100 (Merck) in 1% BSA for 30 minutes. Coverslips were then incubated with primary antibodies in 1% BSA overnight at 4°C. The next day they were washed and treated with secondary antibodies (488 nm and 568 nm) for 75 minutes in the dark at RT. After washing and staining with Hoechst, coverslips were mounted on slides.

For neural stem cell confirmation from hIPSCs, cultures were stained with antibodies against neural stem cell markers Nestin (Abcam, ab22035), Pax6 (BioLegend, 862001), and Sox2 (Abcam, ab97959). Neurons were stained with anti–βIII-tubulin (TUJ1) (Synaptic Systems, 302 306), anti-vGLUT2 (Proteintech, 25261-1-AP), anti–tyrosine hydroxylase (Sigma-Aldrich, ZRB2381), and anti-NeuN (Sigma-Aldrich, MAB377). Imaging was performed using a Leica DM400b epifluorescence microscope, capturing fluorescent signals in FITC, TRITC, and BFP channels. Images were processed with ImageJ version 2.14.0/1.54f (NIH).

#### Primary mouse neuron ICC.

Postsynaptic density was visualized using PSD95 as a marker. To visualize dendritic spines, anti–βIII-tubulin complemented with Alexa Fluor 568 phalloidin to enable visualization of small spines (Invitrogen, A12380) were used. Cultured neurons were first fixed with 4% PFA and permeabilized in blocking buffer (0.1% Triton X-100 in 2.5% BSA-PBS) for 1 hour at RT. Subsequently, neurons were incubated overnight at 4°C with primary antibodies against βIII-tubulin (BioLegend, 801201; 1:500), PSD95 (Abcam, ab18258; 1:200), and m^6^A (Synaptic Systems, 202 003; 1:200). After overnight incubation, neurons were washed in PBS to remove loosely bound antibody and then incubated in species-specific secondary antibodies at RT for 1 hour. Anti–mouse IgG-Cy5 (Invitrogen, A10524; 1:500) and anti–rabbit IgG-FITC (Invitrogen, F-2765; 1:500) were used to bind and visualize primary antibodies. Alexa Fluor 568 phalloidin (Invitrogen, A12380; 1 U/slide) was added to the blocking buffer along with secondary antibodies. Coverslips were mounted using mounting medium with DAPI (Abcam, ab228549) and imaged using a Zeiss LSM 800 confocal microscope.

For PSD95 and dendritic spine quantification, a total of at least 32 dendrites from 22 neurons per treatment group were included in the analysis across a total of 4 experiments. Multiple dendrites sampled from an individual neuron were averaged for statistical analysis to avoid pseudoreplication. Each experiment used embryos from an individual dam (4 in total), the average litter size was 7.75. All imaging and quantifications were performed manually, in a blinded manner with respect to the treatment groups. Images were calibrated for distance per pixel length, and the distance from the soma was measured and divided into 20-μm segments using ImageJ. A total dendritic length of 4440–4500 μm, derived from at least 32 dendrites per treatment group, was analyzed. Images were generated to show the entire neuron using a 40× objective lens. Dendrites, distinct from other dendrites and crossings and extending at least 100 μm from the soma, were captured at ×63 magnification. Utilizing a 2-way repeated-measures (RM) ANOVA, following Tukey’s post hoc multiple-comparison test, all treatment groups along the dendritic distance were analyzed. PSD95 and spine density was also quantified by branch order, using multiple *t* test analysis. A significance level of 0.05 was chosen, and data are expressed as the mean ± SEM. Statistical analysis was performed using GraphPad Prism 8.0 software.

### METTL3 overexpression

hIPSC-derived neurons were transduced with AAV vectors (VectorBuilder, custom-designed) to overexpress human METTL3. Three distinct AAV vectors were used: AAV9-SYN1-EGFP-hMETTL3, which encodes the human METTL3 gene; AAV9-SYN1-EGFP-Empty, a control vector containing only the GFP reporter driven by the SYN1 promoter; and a GFP-only virus without the *SYN1* promoter (negative control). The SYN1 promoter was used to drive neuron-specific expression of both the transgene (hMETTL3) and the GFP reporter, ensuring that the transduction selectively targeted neuronal cells. The GFP reporter enabled the monitoring of successful transduction by providing a fluorescent signal visible under appropriate microscopy conditions.

For the transduction process, hIPSC-derived neurons were cultured as described above for 21 days. The AAV vectors were then added to the culture medium at a concentration of 1 × 10^4^ genomic copies per cell and the neurons were incubated with the vectors for 48 hours, to allow efficient uptake and expression of the transgenes. After incubation, the cells were maintained in fresh culture medium for an additional period to ensure stable transgene expression.

To confirm successful overexpression of METTL3, Western blotting was performed as described above to detect the METTL3 protein in the transduced neurons. The expression of GFP was also evaluated to verify the success of the transduction using GFP immunofluorescence microscopy, as described above. This dual approach allowed for both qualitative and quantitative confirmation of transgene expression. Control cells were similarly transduced with either the empty vector (AAV9-SYN1-EGFP-Empty) or GFP-only vector, and the same analyses were performed to compare the level of GFP expression and METTL3 overexpression across conditions.

### Reagents

All reagents were purchased from commercial sources that are listed throughout. Antibodies used in this study were all purchased from commercial sources. Full Western blot images are provided in supplemental material. Catalog numbers for all antibodies are provided along with vendor information. The detected bands in Western blot analyses correspond to the expected molecular weights of the target proteins, consistent with the manufacturer’s specifications and previous literature where available. Primers were designed in-house using UCSC Genome Browser alignments (mouse, mm39; human, hg38) and NCBI Primer Blast.

### Statistics

Power analyses were performed during the experimental design stage. Effect size was determined depending on the type of downstream analysis based on previous studies along with a significance level of 0.05 and a 1 – β value of 0.8, with equal allocation ratios used throughout.

Unless otherwise stated in specific sections, statistical analyses were performed using appropriate parametric or nonparametric tests based on data distribution. Normality of the data was assessed using the Shapiro-Wilk test. For comparisons between 2 groups, unpaired *t* tests were used when both groups followed a normal distribution with equal variances, while Welch’s *t* test was applied when variances were unequal. If data were not normally distributed, the Mann-Whitney *U* test was used as a nonparametric alternative. For comparisons involving more than 2 groups, 1-way ANOVA was performed for normally distributed data, followed by Tukey’s post hoc test for multiple comparisons, while the Kruskal-Wallis test was used for nonparametric data, followed by Dunn’s test for pairwise comparisons. Statistical significance was set at *P* less than 0.05, and multiple comparisons were corrected using the Benjamini-Hochberg FDR method where applicable. All analyses were conducted using GraphPad Prism or RStudio.

### Study approval

All animal procedures were performed in accordance with the principles of the European Union Directive (2010/63/EU) and were reviewed and approved by the Research Ethics Committee of the Royal College of Surgeons Ireland (REC842) under license from the Health Products Regulatory Authority (HPRA) (AE19127/IO84, AE19127/I152, AE19127/I089) and the UCD Animal Research Ethics Committee (AREC) under license from the HPRA (AE18982/P194). The systemic KA model in rats was performed at Monash University following approval by the Alfred Research Alliance Animal Ethics Committee (E/2058/2020/M). All efforts were made to minimize animal suffering and to use the minimum number of animals required for robust statistical analysis.

The human study was approved by the Ethics Committee at Beaumont Hospital, Dublin (05/18). Written, informed consent was obtained from all individuals participating in the study. All individuals were diagnosed with TLE and underwent surgical tissue resection as part of their treatment.

### Data availability

RNA-seq and m^6^A array datasets are available from the NCBI Gene Expression Omnibus (GEO) under accession numbers GSE295244 (RNA-seq) and GEO GSE297566 (m^6^A array). The MS proteomics data have been deposited to the ProteomeXchange Consortium via the PRIDE (62) partner repository with the dataset identifier PXD064519.

Values for all data points in graphs are reported in the Supporting Data Values file. All other datasets are available upon reasonable request to the corresponding author.

## Author contributions

JM, MMW, SS, LVB, PMCE, MC, DCH, and GPB designed the research studies. JM, MMW, SS, LVB, CT, JK, AH, TA, MKAEA, and MC conducted experiments. JM, MMW, SS, LVB, CT, JK, and MC acquired data. JM, MMW, SS, GL, LVB, CT, JK, MS, AL, MC, and GPB analyzed data. ASR, ZL, YH, AL, ND, JC, FMB, MAF, DFOB, and DCH provided resources. PMCE, EMJM, JCG, DCH, and GPB supervised the study. JM, MMW, SS, and GPB wrote the manuscript. DCH and GPB acquired funding. Co–first authors’ names are listed alphabetically according to their first name.

## Supplementary Material

Supplemental data

Unedited blot and gel images

Supporting data values

## Version 1 07/22/2025

Electronic publication
