# Supplementary material for: N6-methyladenosine (m6A) dysregulation contributes to network excitability in temporal lobe epilepsy
Source: JCI Insight. 2025 Jul 22;10(14):e188612. doi: 10.1172/jci.insight.188612 (PMC12288969; doi:10.1172/jci.insight.188612)
Supplement: Supplemental data [file jciinsight-10-188612-s067.pdf]

Supplemental Materials and Methods

Systemic kainic acid model in rats

Epilepsy was induced in adult male Sprague Dawley rats using a repeated low dose kainic acid model as previously described (1). Rats were randomly allocated to either the KA group or sham group (saline). In the KA cohort, animals received intraperitoneal (IP) injections of 5 mg/kg KA. Seizures were monitored using the Racine scale (2). Repeated doses of 5 mg/kg were administered every 45 minutes until the appearance of self-sustained SE (presence of continuous stage IV-V seizures scored using the standard Racine scale (2, 3)) or the maximum dose of 25 mg/kg was reached. The maximum dose actually administered was 20 mg/kg and the mean number of injections administered was 2.9. Animals were excluded if there was no appearance of self-sustained SE. SE was allowed to progress for 4 hours at which time an IP injection of diazepam (Mayne Pharma, Adelaide, Australia, solvent: 40% propylene glycol, 10% alcohol, 5% sodium benzoate, 1.5% benzyl alcohol)) (5 mg/kg) was administered. Animals were euthanised 12 weeks post SE (a timepoint at which epilepsy is likely to have developed by (3)), the hippocampus was removed on ice and prepared for RNA analysis as described below.

RNA-isolation and analysis

RNA was isolated from both mouse and human tissue using a standard Trizol extraction protocol. Briefly, Trizol (Invitrogen 15596026) was added to each individual tissue sample which was then physically lysed with a pestle. The samples were then centrifuged at 13,000 x g for 10 min at 4°C. The supernatant containing RNA was transferred to a fresh RNase free Eppendorf tube and left to stand at room temperature (RT) for 5 min. After this incubation, chloroform (Sigma C2432) was added and samples were shaken vigorously for 15 sec and then incubated again for 3 min at RT. The samples were then phase separated by centrifugation at 12,000 x g for 15 min at 4°C. The upper aqueous phase was transferred to a fresh tube and mixed with isopropanol (Sigma l9516) and GlycoBlue (Invitrogen AM9515) and incubated at -20°C overnight. The following morning the samples were spun at max speed for up to 30 min to precipitate and pellet the RNA. The supernatant was removed and the remaining RNA pellet was washed with ice cold 75 % ethanol (Sigma E7023). After a brief centrifugation the ethanol was removed and the RNA pellet was air dried at RT and resuspended in RNase-free water. The RNA was quantified using a nanodrop (ThermoFisher Scientific) and only samples which scored between 1.8 and 2.2 for 260/280 ratios were included in our downstream analyses. RNA samples were stored at -80°C until needed.

cDNA synthesis, qPCR, RNA-sequencing

1 µg of RNA was treated with DNase (Invitrogen 18068015) to remove contaminating genomic DNA. Samples were then reverse transcribed using random hexamers (Invitrogen N8080127) and reverse transcriptase (Superscript III, Invitrogen 18080093). To quantify gene expression Sybr-Green (Applied Biosystems 4309155) based qPCR detection was used on an Applied Biosystems Quantstudio 7 Real Time PCR machine. Primers used are listed in Table 2.

M^{6}A quantification assay

Colorimetric antibody-based quantification of m^{6}A from total or polyA-RNA was carried out on RNA extracted from either mouse or human tissue using the EpiQuik m^{6}A RNA methylation quantification kit (EpigenTek P-9005) according to the manufacturer's instructions. 200 ng of RNA from each sample was used as starting material and assays were performed in triplicate. Positive and negative controls supplied with the kit were also run in parallel. The RNA from each sample was bound to the well using the binding solution and a 90 minute incubation at 37ºC. Binding solution was then removed and the capture m^{6}A antibody was added to each well and incubated at RT for 60 min. This was followed by the addition of the detection antibody for 30 mins and addition of an enhancer solution. The enhancer solution was removed after 30 mins and after several washes the developing solution was added and the plate absorbance values were read at 450 nm on a plate reader (Clariostar Plus). Relative quantification of each sample was then calculated by comparing to a standard curve generated using the kit supplied positive control RNA.

Western blotting

Whole hippocampal tissue from mice, and ~30 mg human tissue were physically homogenised in a lysis buffer containing 0.01 M Tris-HCl (Sigma T3253) and 1 mM EDTA (Sigma E7889). Protein lysate concentrations were quantified using a BCA assay (Pierce ThermoFisher Scientific 23225). 20 to 50 µg of protein was mixed with 6X loading buffer (New England Biolabs B7021S), denatured and then used immediately or stored at -20°C until use. Protein samples were separated by SDS-polyacrylamide gel electrophoresis (SDS-PAGE) and transferred to either nitrocellulose (ThermoFisher Scientific 88018) or methanol-activated PVDF membrane (ThermoFisher Scientific 88585) by semi-dry (up to 120 kDa)

or wet transfer (over 120kDa) depending on protein size. Membranes were then blocked in 5% milk in PBS (w/v) (Sigma 70166) and incubated with the following primary antibodies: METTL3 (Abcam ab195352), METTL14 (Abcam ab98166), WTAP (Santa-Cruz Biotech sc-374280), YTHDF1 (Proteintech 24744-1-AP), YTHDF2 (Proteintech 24744-1-AP), FTO (Proteintech 27226-1-AP), ALKBH5 (Proteintech 16837-1-AP), β-Actin (Sigma-Aldrich A5441) and GAPDH (Invitrogen AM4300). Membranes were next incubated with HRP-conjugated secondary antibodies (1:1,000-5,000) and bands were visualised using HRP-substrate (Millipore WBKLS0500) on a FujiFilm LAS-4000 system under chemiluminescence.

Induced pluripotent stem cell (IPSC) cultures

A commercially available human induced pluripotent stem cell (hIPSC) line (HPSI0114i-eipl_1, HipSci, donor: female 40-44) (Generated by Wellcome Sanger Institute, supplied by European Bank for Induced Pluripotent Stem Cells) with no known neurological condition was used to generate IPSC-derived neurons. IPSCs were cultured on dilute vitronectin (Gibco A14700)-coated hydrophilic culture plates (Sarstedt) in either Complete Essential 8 (Gibco A1517001) or Essential 8 Flex medium (Gibco A2858501). Cell dissociation for passaging was done by adding prewarmed 0.5 mM EDTA (Invitrogen AM9262) in PBS without calcium or magnesium for 7 min, after washing cells once with prewarmed PBS. Growth medium was changed daily prior to initiation of neural induction. IPSC-derived neurons were cultured in a complete Terminal Differentiation Medium (cTDM) composed of Neurobasal Medium (Gibco 21103049) with Penicillin – Streptomycin (P/S) (Gibco 15070063), Glutamax-100x (Gibco 35050061), DMEM/F-12 (Gibco A4192101) supplemented with N2 (Gibco 17502048), Culture 1 supplement (Gibco A3320201), B27 (Gibco 17504044), BDNF (Merck B3795) and GDNF (Merck G1777) . Neurons were cultured on 1:40 Geltrex (Gibco A1413201) coated acid etched circular glass or plastic cover slips in ibidi microscopy plastic cell culture plates. Media was 50 % changed every 2/3 days depending on growth rate and cells were cultured and matured for between 29 and 30 days. Cells were checked daily and imaged via brightfield microscopy at a magnification range of 40 x – 400 x (Nikon Eclipse TS100).

Primary hippocampal neuron cultures

Primary hippocampal neuronal cultures were prepared following previously described procedures (4). Briefly, brains from embryonic day 18 (E18) pups were removed in a sterile culture hood and immersed in a solution of HBSS without calcium and magnesium to prevent enzymatic degradation and maintain tissue integrity (Corning 20-021-CV). The hippocampi were isolated using fine forceps and disaggregated by finely mincing the tissue with a scalpel and forceps. After mincing, the tissue was transferred to a tube and dissociated from tissue matrices using 0.25% Trypsin-EDTA (1x) (Gibco 25200056) for 15 min at 37ºC. The dissociated cell suspension was gently triturated using a sterile

pipette to dissociate any remaining tissue aggregates. The cells were then counted using a haemocytometer and plated on 16 mm coverslips which had been precoated with 1 mg/ml poly-L-lysine (Sigma P8920) and 20 µg/ml Laminin (Sigma L2020). Cultures were maintained in neurobasal medium (Gibco 21103049) supplemented with B-27 (Gibco 17504044) and N2 (Gibco 17502048) at 37 ºC with 5% CO_2 for 8 to 9 days with media changes every 2 – 3 days. Primary neurons were then treated with 10 µM of the selective METTL3 inhibitor STM2457(Insight Biotechnology HY-134836) which selectively binds and inhibits METTL3 over METTL14 and other methylation-associated enzymes (5) or Veh (1% DMSO in PBS) for control for 24 hours.

Electrophysiology and calcium imaging

To characterise neural stem cell-derived (NSC-derived) neurons differentiated for 4–8 weeks in vitro we used patch-clamp experiments. Spontaneous action potentials were recorded in loose-patch configuration with a Multiclamp 700 B amplifier (Molecular Devices, Ca, USA) , interfaced by an A/D converter (Digidata 1550B, Molecular devices Ca, USA) to a computer using pClamp software (Version 11, Molecular Devices, Ca, USA). Pipette electrodes (Hardward Apparatus, Ma, USA, G150T-4), were fabricated using a vertical puller (Narishige PC-100, Tokyo, Japan). Signals were low-pass filtered at 2 kHz or 10 kHz and sampled at 10 kHz or 50 kHz. Recordings were performed at 32°C in a bath solution containing 135 mM NaCl (Sigma 71386), 3 mM KCl (Sigma P9541), 2 mM $CaCl_2$ (Sigma 21115), 1 mM $MgCl_2$ (Invitrogen AM9530G), 10 mM HEPES (Sigma H0887), and 10 mM glucose (Sigma G7021) (pH 7.2; osmolality 290–300 mmol/kg), and the pipettes were filled with the same solution.

For calcium imaging, cells were loaded with Cal-520 dye (AAT Bioquest, Ca, USA 21130) at 37°C, by incubating with the acetoxymethyl (AM) ester form of the dye at a final concentration of 2 µM in culture media. The dyes were prepared as 5 mM stock solutions in DMSO and kept at -20ºC until the day of use and then diluted fresh. After 45 min, cells were washed several times with dye-free HEPES-buffered saline solution and transferred to an imaging chamber on a microscope (Zeiss Axio Examiner, Jena Germany) equipped with a Zeiss 40x water immersion lens. Zen Blue imaging software (Carl Zeiss, Jena Germany) was used for hardware control and image acquisition, and ImageJ for analysis. Imaging was performed at 32°C in a bath solution containing 135 mM NaCl (Sigma 71386), 3 mM KCl (Sigma P9541), 0.1 mM $CaCl_2$ (Sigma 21115), 0 mM $MgCl_2$, 10 mM HEPES (Sigma H0887), and 10 mM glucose (Sigma G7021) (pH 7.2; osmolality 290–300 mmol/kg), with images acquired at 2 Hz. Background fluorescence was measured outside the soma of interest in each frame of every time series. Regions of interest were manually drawn around the soma and baseline fluorescence intensity (F0) was determined by averaging 14 frames preceding the cells exposure to BzATP or ATP and the time course of normalised fractional dye fluorescence [ΔF/F0] was obtained where ΔF equals F(t) - F0.

METTL3 overexpression

IPSC-derived neurons were transduced with Adeno-associated virus (AAV) vectors (VectorBuilder, custom-designed) to overexpress human METTL3. Three distinct AAV vectors were used: AAV9-SYN1-EGFP-hMETTL3, which encodes the human METTL3 gene, AAV9-SYN1-EGFP-Empty, a control vector containing only the GFP reporter driven by the SYN1 promoter and a GFP only virus without the SYN1 promoter (negative control). The SYN1 promoter was used to drive neuron-specific expression of both the transgene (hMETTL3) and the GFP reporter, ensuring that the transduction selectively targeted neuronal cells. The GFP reporter enabled the monitoring of successful transduction by providing a fluorescent signal visible under appropriate microscopy conditions.

For the transduction process, IPSC-derived neurons were cultured as described above for 21 days. The AAV vectors were then added to the culture medium at a concentration of 1 x 10^4 genomic copies per cell and the neurons were incubated with the vectors for 48 hours, to allow efficient uptake and expression of the transgenes. After incubation, the cells were maintained in fresh culture medium for an additional period to ensure stable transgene expression.

To confirm successful overexpression of METTL3, western blotting was performed as described above to detect the METTL3 protein in the transduced neurons. The expression of GFP was also evaluated to verify the success of the transduction using GFP immunofluorescence microscopy as described above. This dual approach allowed for both qualitative and quantitative confirmation of transgene expression. Control cells were similarly transduced with either the empty vector (AAV9-SYN1-EGFP-Empty) or GFP-only vector, and the same analyses were performed to compare the level of GFP expression and METTL3 overexpression across conditions.

Cellular bioenergetics assay and analysis

A Seahorse XFe96 analyser (Agilent technologies) was used to determine the real time levels of oxygen consumption (OCR) and extracellular acidification rate (ECAR). Neural stem cells were plated directly onto Geltrex (Gibco A1413201)-coated Agilent Seahorse FX96 well plates at a concentration of ~5 x 10^5 cells / well. Neurons were grown for 28 days post terminal differentiation. Cells were assigned to one of four treatment groups; METTL3 OE, CtrlVector, No Treatment, and METTL3 Inhibitor (STM2457, 10 µM). Treatment and control AAVs were added to the plated cells 7 days prior to analysis. The METTL3 inhibitor was freshly prepared and added to one group of cells at a concentration of 10 µM 24h prior to analysis. On the day of analysis cells were washed with assay medium (unbuffered DMEM supplemented with 10 mM glucose, pH 7.4) and incubated for 30 min at 37 ºC in a non-CO_2 incubator. The basal oxidative phosphorylation/glycolysis was calculated from the average of 3 baseline OCR/ECAR measurements obtained prior to injection of specific metabolic inhibitors; oligomycin

(ATP synthase-inhibitor, 2 μg/ml, Agilent), FCCP (mitochondrial uncoupler, 5 μM, Agilent) and antimycin A (complex-III inhibitor, 2 μM, Agilent) to measure both the maximal glycolytic rate and ATP synthesis, the maximal respiratory capacity and respiratory reserve respectively. Following completion of the Seahorse assay the plate was removed and the media removed from each well. Protein lysis buffer was then added and protein levels per well were determined using a BCA assay as described above for normalisation and to control for variation in cell numbers in each individual well. Assay was repeated 3 times on new differentiations

Supplementary Figure 1

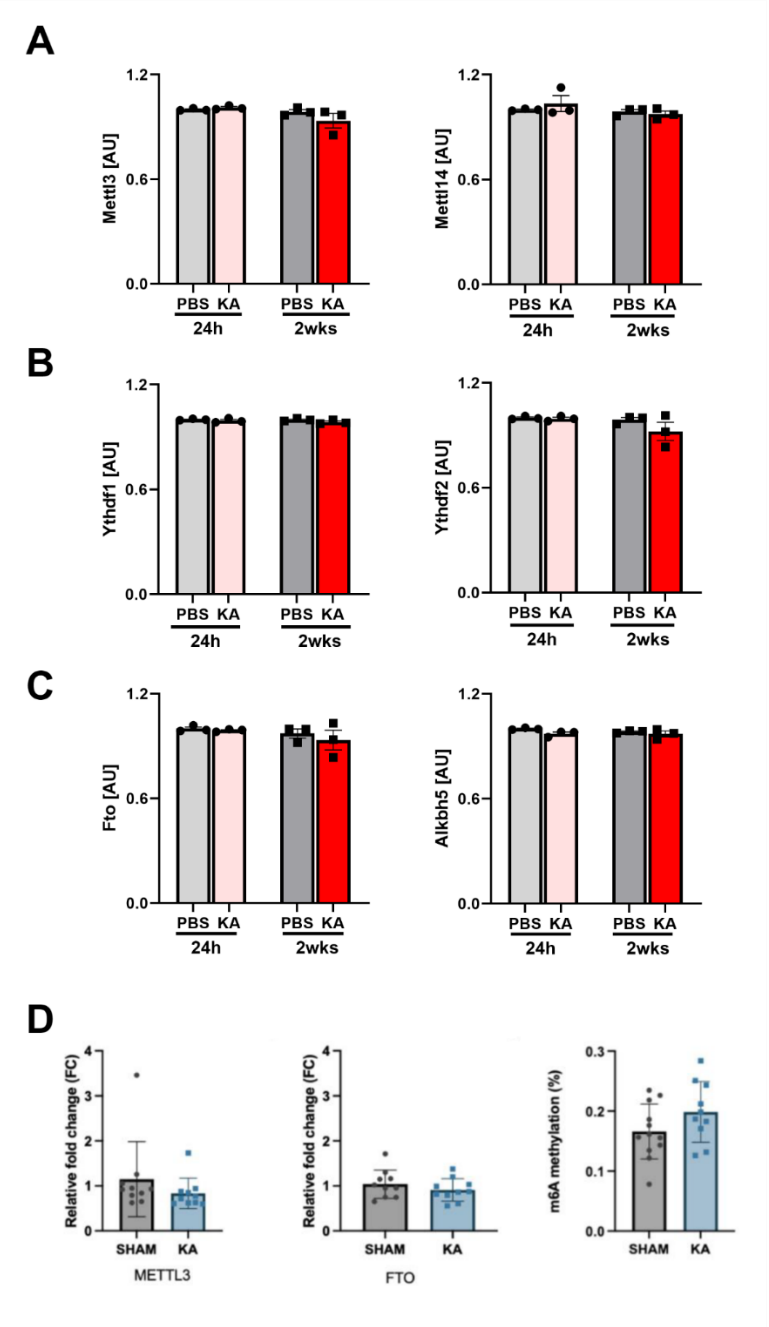

Supplementary Figure 1: qPCR analysis of m^{6}A-associated proteins in experimental epilepsy

(A - C) qPCR analysis of m^{6}A writers METTL3 and METTL14 **(A),** readers YTHDF1 and 2 **(B)** and erasers FTO and ALKBH5 **(C)** from hippocampal tissue from acute (24h) and chronic mice (2wks). **(D)** Relative quantification of MTLL3, FTO and total m^{6}A abundance in hippocampus from sham and systemic KA treated rats 12 weeks post SE. (Unpaired t-tests)

Supplementary Figure 2

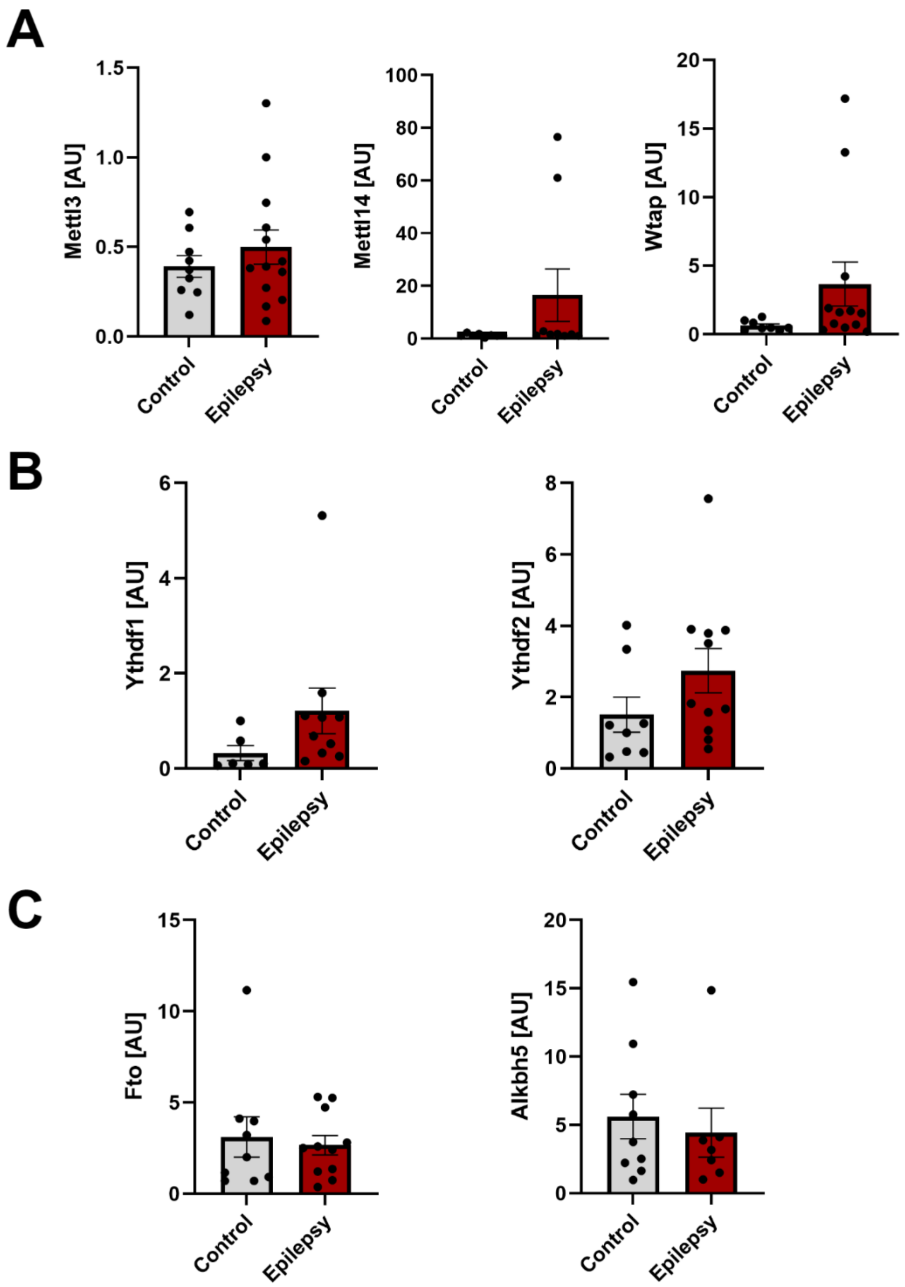

Supplementary Figure 2: qPCR analysis of m^{6}A-associated proteins in human TLE

(A - C) qPCR analysis of m^{6}A writers METTL3, METTL14 and WTAP **(A),** readers YTHDF1 and 2 **(B)** and erasers FTO and ALKBH5 **(C)** from hippocampal tissue from patients with drug refractory TLE compared to age and sex-matched autopsy control tissue. (Unpaired t-tests or Mann-Whitney tests)

Supplementary Figure 3

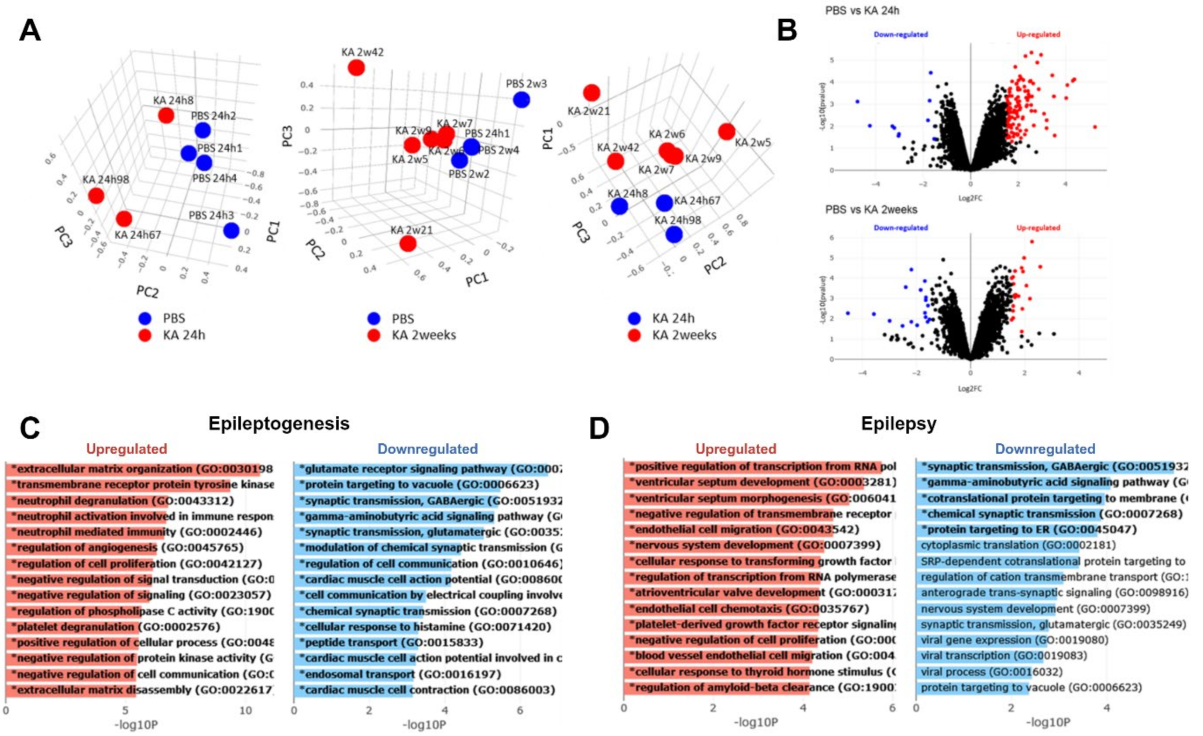

Supplementary Figure 3: RNA-sequencing of experimental epilepsy using the intra-amygdala kainic acid model.

(A) Principle component analysis of PBS and KA treated animals at 24h and 2 weeks post status epilepticus. **(B)** Volcano plot representation of differential gene expression in epileptogenic and epileptic mice compared to time-matched PBS controls. **(C-D)** Gene ontology analysis of enriched pathways in epileptogenic and epileptic mice.

Supplementary Figure 4

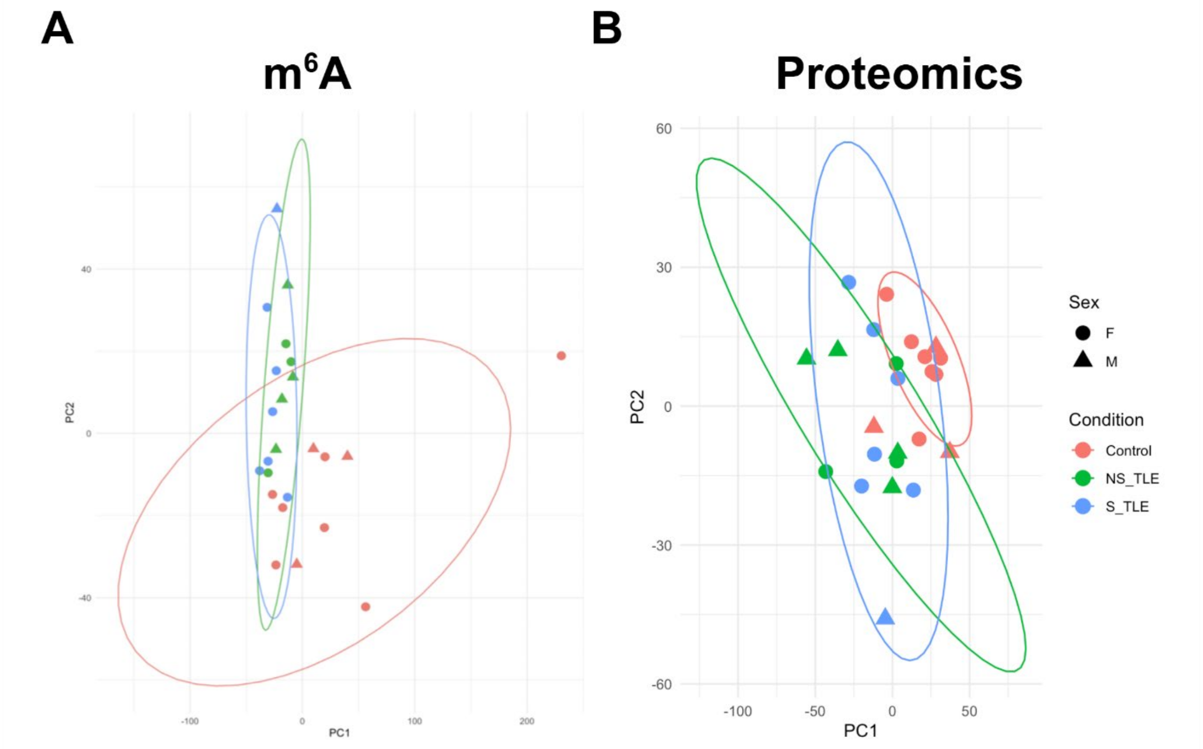

Supplementary Figure 4: Proteomic analysis of human TLE

(A) Principal component analysis of human m6A profiling based on condition and sex. (B) Principal component analysis of proteomic profiling using mass spectrometry on control, human TLE with (S) and without sclerosis (NS) and sex as a variable. Legend applicable to both plots.

Supplementary Figure 5

log2FoldChange	pvalue	Transcript name	gene_biotype
-4,7858441	0,029071522	phosphoribosylaminoimidazole carboxylase, phosphoribosylaminoimidazole succinocarboxamide synthetase pseudogene 3	processed_pseudogene
-4,6538890	0,007351171	novel transcript	antisense
-4,4382630	0,01016981	novel transcript	lincRNA
-4,3429562	0,002859274	long intergenic non-protein coding RNA 1535	lincRNA
-4,3183999	0,015107362	novel transcript	lincRNA
-4,1900696	0,037441989	DnaJ (Hsp40) homolog, subfamily C member 8 (DNAJC8) pseudogene	processed_pseudogene
-4,1177165	0,030079106	ribosomal protein L10 (RPL10) pseudogene	processed_pseudogene
-4,1176378	0,00520724	histone cluster 1 H3 family member j	protein_coding
-4,1102012	0,036628714	golgin A6 family member D	protein_coding
-4,0603839	0,016824913	novel transcript, antisense to SGIP1	antisense
-4,0458461	0,04303308	RNA, U6 small nuclear 1278, pseudogene	snRNA
-4,0309379	0,038790486	chromosome 16 open reading frame 78	protein_coding
-3,9746342	0,046145674	ribosomal protein S27a (RPS27A) pseudogene	processed_pseudogene
-3,5554791	0,033683351	TEC	TEC
-3,5098987	0,026765531	microRNA 3180-4	miRNA
-3,4123093	0,043255742	novel transcript, antisense CCL3L3	antisense
-3,3760628	0,037141339	RNA, U7 small nuclear 3 pseudogene	snRNA
-3,3573019	0,042054287	La ribonucleoprotein domain family member 1 pseudogene 1	processed_pseudogene
-3,3085312	0,049797988	novel transcript	antisense
-3,2999565	0,029475971	small nucleolar RNA, H/ACA box 31	snoRNA

Supplementary Figure 5: Pseudogenes and non-coding RNAs are decreased in hIPSC-derived neurons when METTL3 is overexpressed

List of top 20 most downregulated transcripts when METTL3 is overexpressed in hIPSC-derived neurons.

Supplementary Figure 6

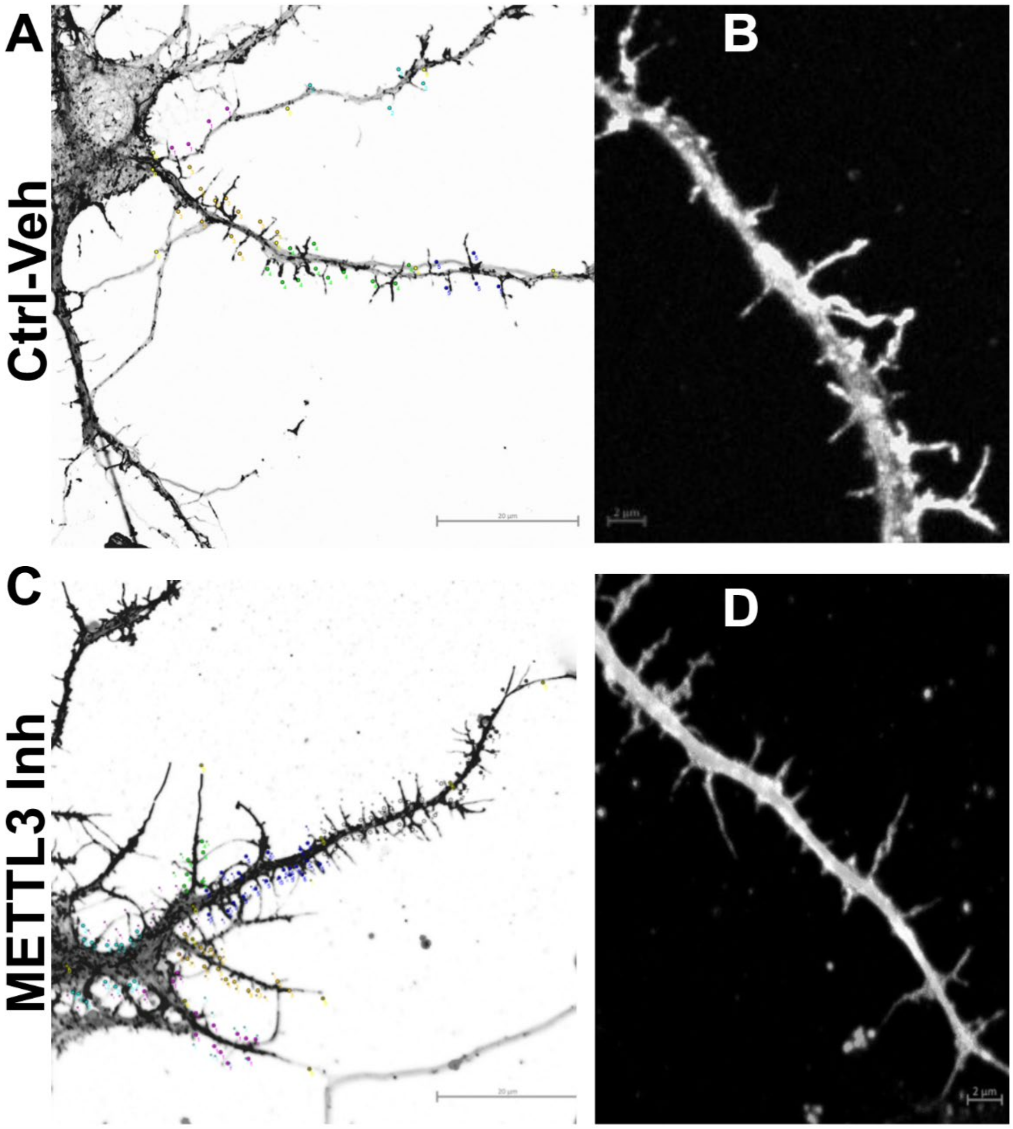

Supplementary Figure 6:

A, C) Representative Confocal microscope images depicting low magnification soma and dendrite marked up for dendritic spine quantification from Veh treated (A) and METTL3 Inh treated (B) cells, **B)** high magnification of individual dendrite and spines from neurons depicted in A and C.

Supplementary Figure 7

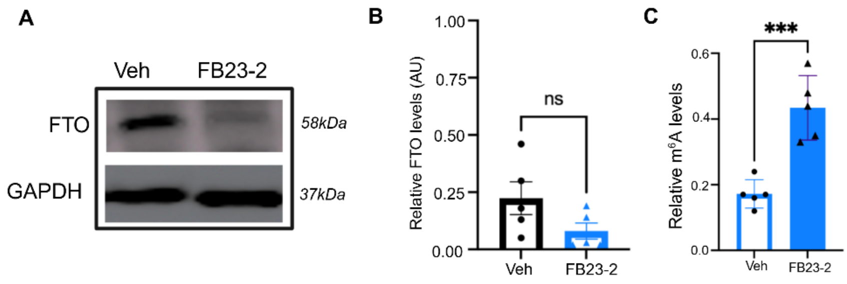

Supplementary figure 7: (A, B) Western blot and densitometric quantification of FTO protein levels in the hippocampus 24h post Veh or FB23-2 FTO inhibitor (n = 6/group) (Unpaired t-test t = 1.801, df = 8, p = 0.1095).

(C) m^{6}A quantification using colorimetric assays to quantify hippocampal m^{6}A levels from naïve mice treated with either Veh or FB23-2 (measurement taken 24h post drug treatment) (n = 6/group) (Unpaired t-test t = 5.463, df = 8, p = 0.0006).

Supplementary Figure 8

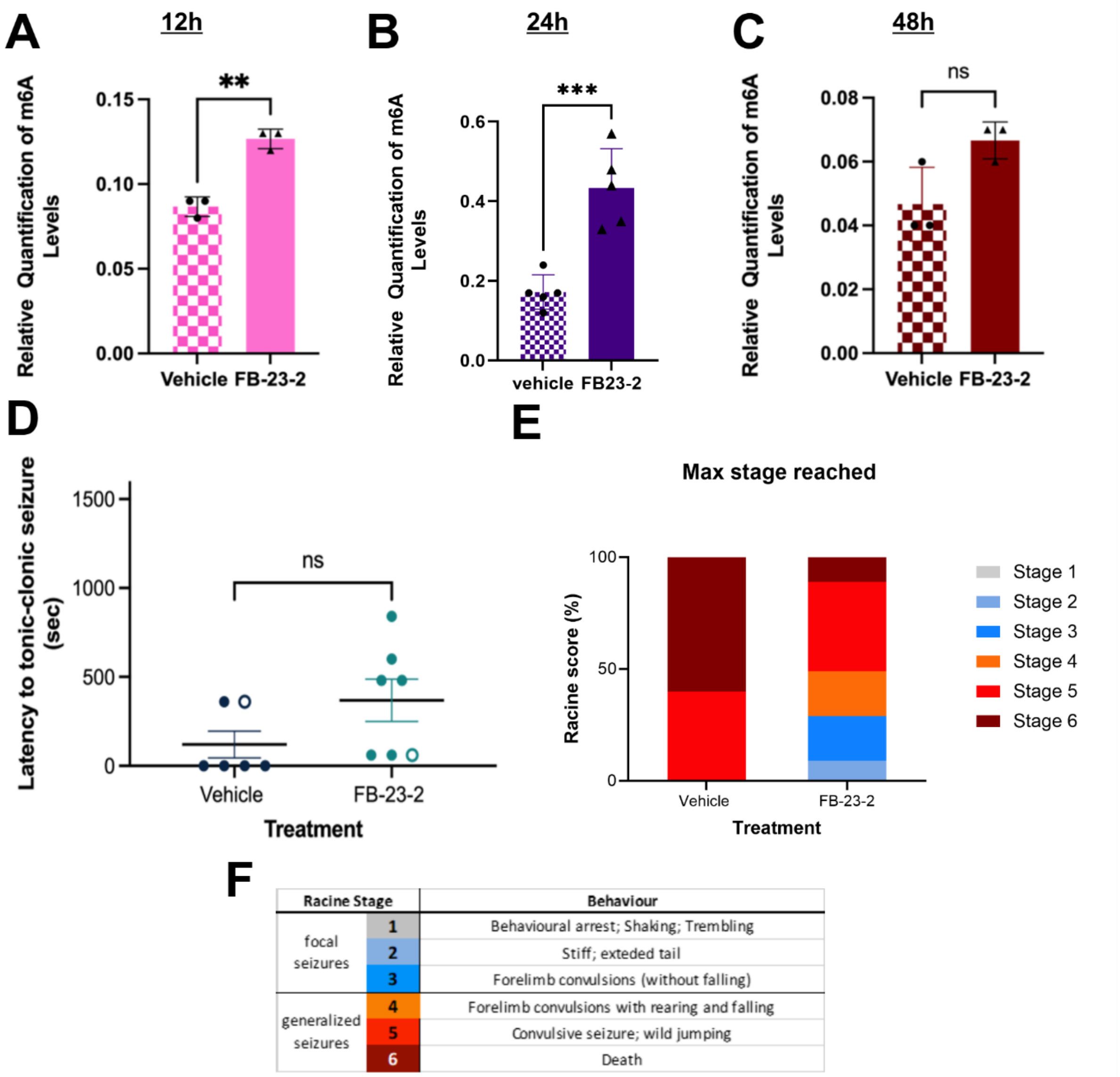

Supplementary Figure 8:

(A – C) Colorimetric quantification of m^{6}A levels in hippocampal tissue 12, 24 and 48 hours following a single 20 μM administration of FB23-2. **(D)** Time to onset of seizure activity following sub-cutaneous PTZ administration. **(E)** Maximum Racine score reached in Veh and FB23-2 treated mice over the course of 25 minute recording period post PTZ administration. **(F)** Adapted Racine scale used to score PTZ-induced seizures. (A-D Unpaired t-tests, E: Chi square)

Supplementary Table 1: Human sample information

Table 1: List of human samples and characteristics/diagnosis

Sample ID	Sex	Age (years)	Onset (years)	Diagnosis	Neurosurgical Procedure	Failed/discontinued ASMs	ASMs at time of surgery
**BH028**	F	42	15	Non Sclerotic (TLE)	Right sided temporal lobectomy	CBZ, LCM, LEV, PHT, VPA, ZNS	CLB, LTG, OXC, PER
**BH035**	M	56	30	Non Sclerotic (TLE)	Right sided temporal lobectomy	CBZ, LTG, LEV, TPM	CLB, LCM, OXC
**BH093**	M	22	15	Non-Sclerotic (TLE)	Left sided temporal lobectomy	OXC, ZNS	LCM, LEV
**BH101**	F	43	11	Non-Sclerotic (TLE)	Left sided temporal lobectomy	LTG, LEV, VGB	CBZ, LCM
**BH116**	M	39	7	Non-Sclerotic (TLE)	Right sided temporal lobectomy	CBZ, LEV, VPA	CLB, LTG, ZNS
**BH159**	M	55	10	Non-Sclerotic (TLE)	Left sided temporal lobectomy	UNKOWN	ESL, LTG, VPA
**BH161**	F	34	32	Non-Sclerotic (TLE)	Right sided temporal lobectomy	Nil	LCM, LEV, MID

**BH027**	F	27	1	Sclerotic (TLE)	Right sided temporal lobectomy	ACT, BRV, CBZ, CLN, GBP, LCM, LTG, LEV, LZP, OXC, PB, TPM, VPA, ZNS	CLB, DZP, MID, TGB
**BH029**	F	25	10	Sclerotic (TLE)	Left sided temporal lobectomy	CBZ, GBP, TPM	LCM, LEV, TPM
**BH046**	M	43	17	Sclerotic (TLE)	Right sided temporal lobectomy	OXC, ZNS, LEV, PGB, TGB, TPM, ZNS	CBZ, CLB, ESL, GBP, LTG, MID, PHT
**BH129**	F	46	31	Sclerotic (TLE)	Right sided temporal lobectomy	CBZ, LTG, OXC	LCM, LEV, MID
**BH131**	F	54	16	Sclerotic (TLE)	Right sided temporal lobectomy	CBZ	CLB, LTG, LEV
**BH132**	F	60	22	Sclerotic (TLE)	Right sided temporal lobectomy	LEV	CBZ, GBP, LTG, VPA
**BH144**	F	38	2	Sclerotic (TLE)	Right sided temporal lobectomy	CBZ, ESL, LCM, PB, PHT	LEV, PER
**329**	M	18		Unaffected Autopsy Control			

**1156**	F	45	Unaffected Autopsy Control
**5337**	F	28	Unaffected Autopsy Control
**5615**	M	50	Unaffected Autopsy Control
**5644**	F	29	Unaffected Autopsy Control
**5646**	F	20	Unaffected Autopsy Control
**5751**	F	25	Unaffected Autopsy Control
**5844**	F	42	Unaffected Autopsy Control
**5917**	M	49	Unaffected Autopsy Control
**6235**	F	21	Unaffected Autopsy Control

Abbreviations: ACT, acetazolamide; ASMs, antiseizure medicines; BRV, brivaracetam; CBZ, carbamazepine; CLB, clobazam; DZP, diazepam; ESL, eslicarbazepine; F, female; GBP, gabapentin; LCM, lacosamide; LEV, leviteracetam; LTG, lamotrigine; LZP, lorazepam; M, male; MID, buccal midazolam; OXC, oxcarbazepine; PB, phenobarbital; PER, perampanel; PGB, pregabalin; PHT, phenytoin; TGB, tiagabine; TPM, topiramate; VPA, valproate; VGB, vigabatrin; ZNS, zonisamide

Supplementary Table 2: Primers

qPCR			
**Gene**	**Species**	**Fw (5' – 3')**	**Rv (5' – 3')**
**Mettl3**	Mse	ACATCTGTGGCCCCTGAACTA	TGGCGTAGAGATGGCAAGAC
**Mettl3**	Hsa	ATTTTCCGGTTAGCCTTCGGGG	TAGCTTACAGAGCCATGGCT
**Mettl14**	Mse	GCTAAGTCAAACACTCCTCCCA	TATTCTTCCAGAGGGGGCTC
**Mettl14**	Hsa	AAGTCTCTACTGAGGAAAGCTA	ATTAAGGTACCCACCACAATAC
**Wtap**	Mse	GCCCCAACGTTTAAGTGCAG	AGCATTCGACACTTCGCCAT
**Wtap**	Hsa	GCCCCAACGTTTAAGTGCAG	AGCATTCGACACTTCGCCAT
**Ythdf1**	Mse	CTGCAGTTAAGACGGTGGGT	TAGCAATGGCTGCCCATGAA
**Ythdf1**	Hsa	ATTGGAGTCGACGCCTCCTCA	AGGCTTGTATTCTCCTAGAGG
**Ythdf2**	Mse	CTGCTGTTGGTAGTGGGTCC	GGCTGTTGTTTTGCAGGCTT
**Ythdf2**	Hsa	AGAGCGTCGCCGAGTCGGA	TGATGCTCAACAGGATCTC
**FTO**	Mse	GTGTTTTGGCTGGCTCACAG	GTCGCCATCGTCTGAGTCAT
**FTO**	Hsa	CTACGCTCTTCCAGCTGTCGGA	TCGGTTTCTTATCTCATCCT
**Alkbh5**	Mse	TAGATGCACCGCGATTGGAA	CTCATCTTCACCTTGCGGGT
**Alkbh5**	Hsa	GAGGAGCCCGCTAAGGAGCGGC	TGTAGAGCACACATATCAGGGC
**Gapdh**	Mse	AGGTCGGTGTGAACGGATTTG	TGTAGACCATGTAGTTGAGGTCA
**Gapdh**	Hsa	AGCCACATCGCTCAGACAC	GCCCAATACGACCAAATCC

Supplementary Table 3 Statistical analysis summary Fig 4D and 4E

Gene Name	Test	U Value	P Value	T value	DF Value	M6A status
**Mier 1**	Unpaired t-test		0.0001	16.19	7	Hyper
**Serpina3n**	Unpaired t-test		0.021	2.893	8	Hyper
**Dido 1**	Unpaired t-test		0.0547	2.248	8	NS
**PI3Kr1**	Unpaired t-test		0.01	3.355	8	Hypo
**Atp2B3**	Unpaired t-test		0.0135	3.153	8	Hypo
**ATF7IP**	Unpaired t-test		0.0493	2.26	8	Hypo
**Serpina3n**	Unpaired t-test		0.0001	6.727	8	Hyper
**Pcdhgc4**	Mann-Whitney	5	0.1508			NS
**Zfp57**	Mann-Whitney	0	0.0159			Hyper
**OPRK1**	Unpaired t-test		0.0081	3.5	8	Hypo
**Mastl**	Unpaired t-test		0.014	3.131	8	Hypo
**Lrrc20**	Unpaired t-test		0.0875	1.946	8	NS

References:

1. Brennan GP, Dey D, Chen Y, Patterson KP, Magnetta EJ, Hall AM, et al. Dual and Opposing Roles of MicroRNA-124 in Epilepsy Are Mediated through Inflammatory and NRSF-Dependent Gene Networks. *Cell Rep.* 2016;14(10):2402-12.
2. Racine RJ. Modification of seizure activity by electrical stimulation. II. Motor seizure. *Electroencephalogr Clin Neurophysiol.* 1972;32(3):281-94.
3. Casillas-Espinosa PM, Anderson A, Harutyunyan A, Li C, Lee J, Braine EL, et al. Disease-modifying effects of sodium selenate in a model of drug-resistant, temporal lobe epilepsy. *Elife.* 2023;12.
4. Engel T, Brennan GP, Sanz-Rodriguez A, Alves M, Beamer E, Watters O, et al. A calcium-sensitive feed-forward loop regulating the expression of the ATP-gated purinergic P2X7 receptor via specificity protein 1 and microRNA-22. *Biochim Biophys Acta Mol Cell Res.* 2017;1864(2):255-66.
5. Yankova E, Blackaby W, Albertella M, Rak J, De Braekeleer E, Tsagkogeorga G, et al. Small-molecule inhibition of METTL3 as a strategy against myeloid leukaemia. *Nature.* 2021;593(7860):597-601.
