# Supplementary material for: N6-methyladenosine (m6A) dysregulation contributes to network excitability in temporal lobe epilepsy
Source: JCI Insight. 2025 Jul 22;10(14):e188612. doi: 10.1172/jci.insight.188612 (PMC12288969; doi:10.1172/jci.insight.188612)

# Figure 1

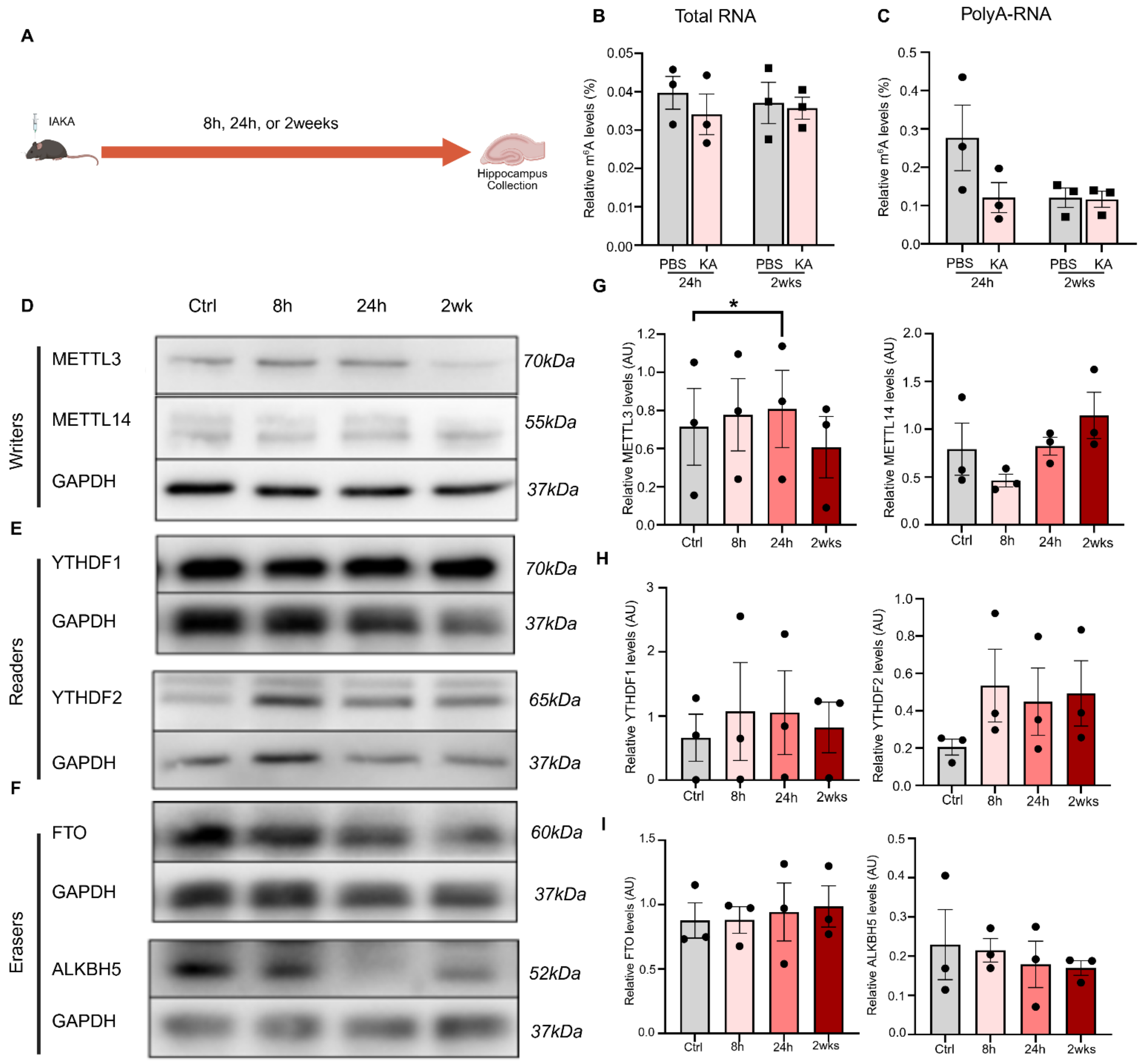

A
IAKA
8h, 24h, or 2weeks
Hippocampus Collection
B
Total RNA
Relative m6A levels (%)
PBS KA
24h
2wks
C
PolyA-RNA
D
Ctrl 8h 24h 2wk
Writers
METTL3 70kDa
METTL14 55kDa
GAPDH 37kDa
E
Readers
YTHDF1 70kDa
GAPDH 37kDa
YTHDF2 65kDa
GAPDH 37kDa
F
Erasers
FTO 60kDa
GAPDH 37kDa
ALKBH5 52kDa
GAPDH 37kDa
G
Relative METTL3 levels (AU)
Relative METTL14 levels (AU)
Ctrl 8h 24h 2wks
H
Relative YTHDF1 levels (AU)
Relative YTHDF2 levels (AU)
I
Relative FTO levels (AU)
Relative ALKBH5 levels (AU)

# Figure 1D

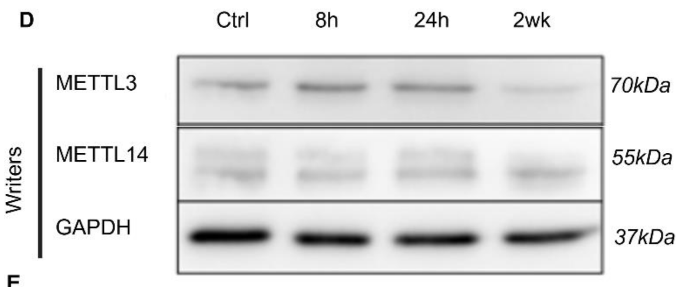

D
Ctrl
8h
24h
2wk
METTL3
METTL14
GAPDH
Writers
70kDa
55kDa
37kDa


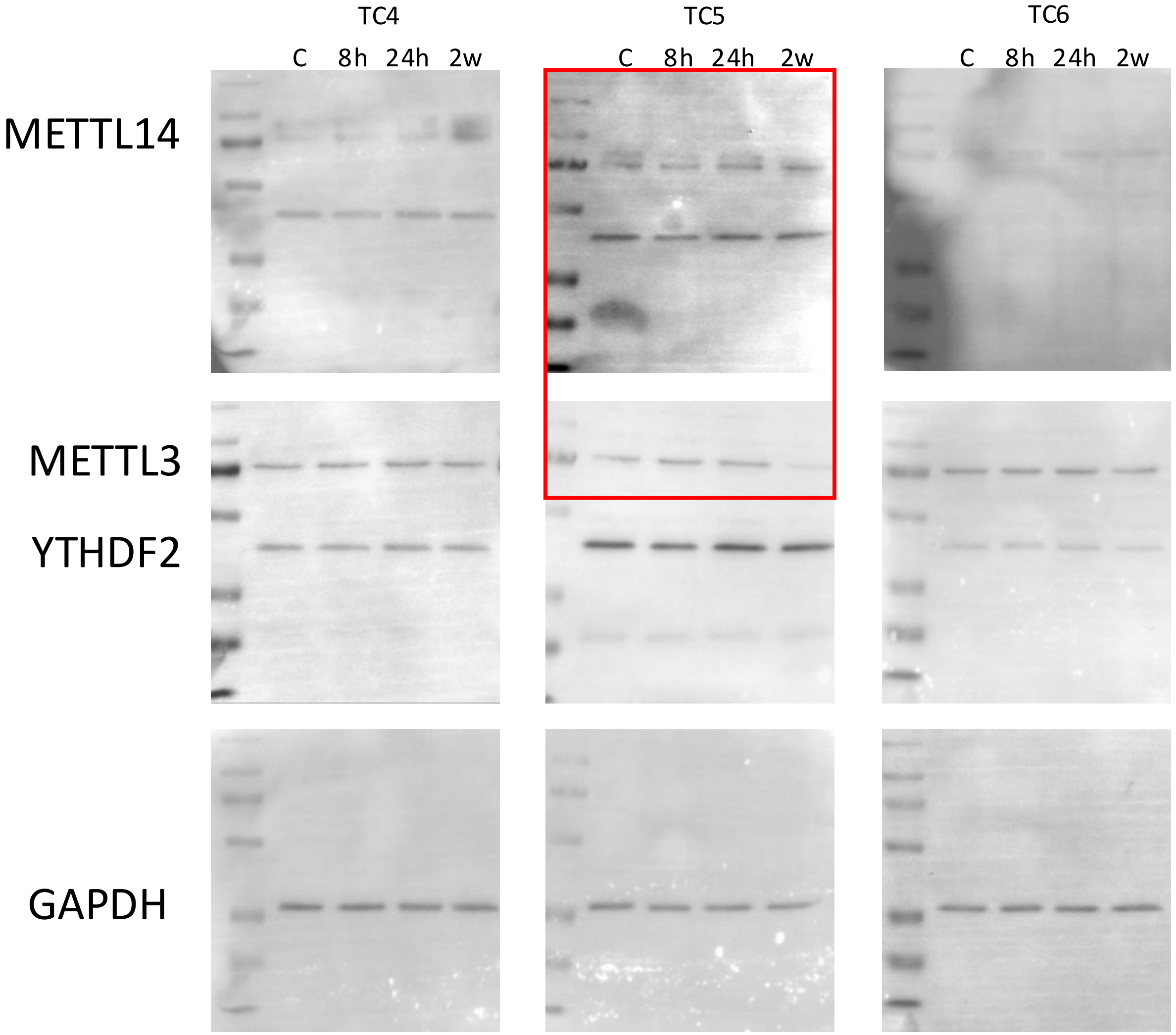

TC4
TC5
TC6
C 8h 24h 2w
C 8h 24h 2w
C 8h 24h 2w
METTL14
METTL3
YTHDF2
GAPDH

## Figure 1E

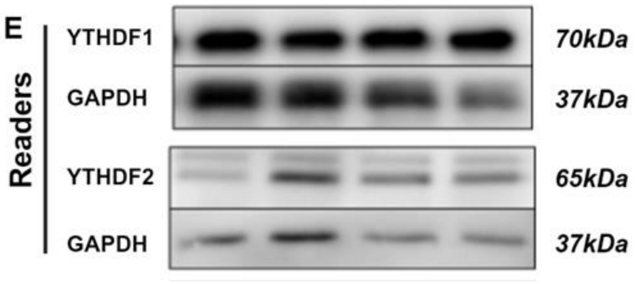
E
Readers
YTHDF1
GAPDH
YTHDF2
GAPDH
70kDa
37kDa
65kDa
37kDa

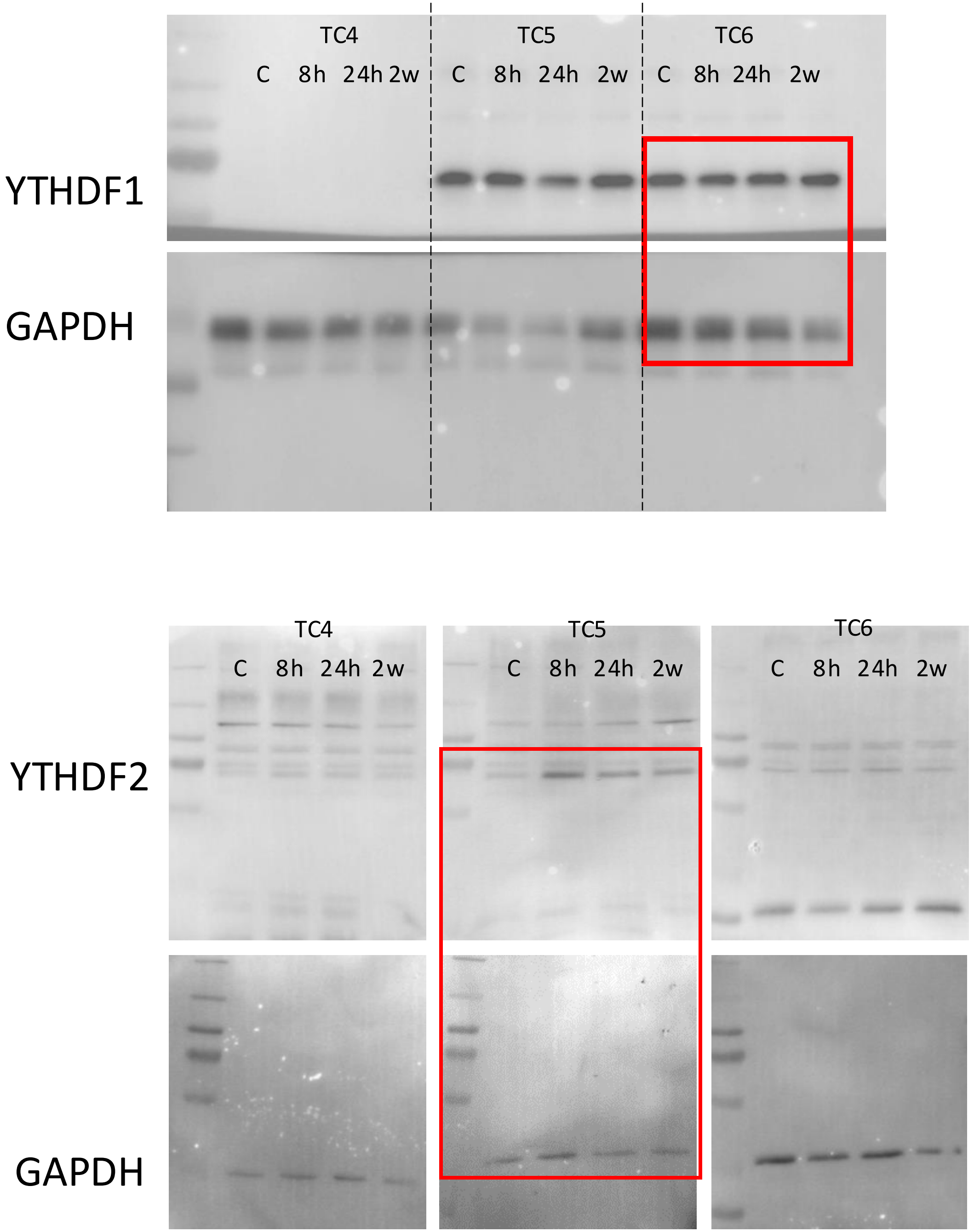
TC4
TC5
TC6
C 8h 24h 2w
C 8h 24h 2w
C 8h 24h 2w
YTHDF1
GAPDH
TC4
TC5
TC6
C 8h 24h 2w
C 8h 24h 2w
C 8h 24h 2w
YTHDF2
GAPDH

## Figure 1F

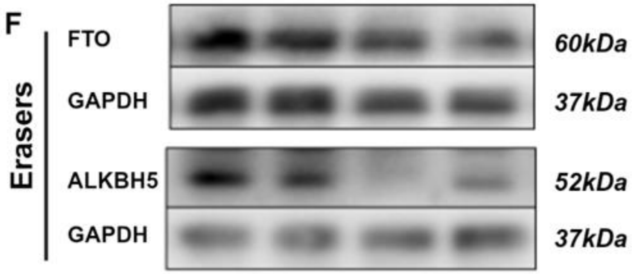

F
Erasers
FTO
GAPDH
ALKBH5
GAPDH
60kDa
37kDa
52kDa
37kDa


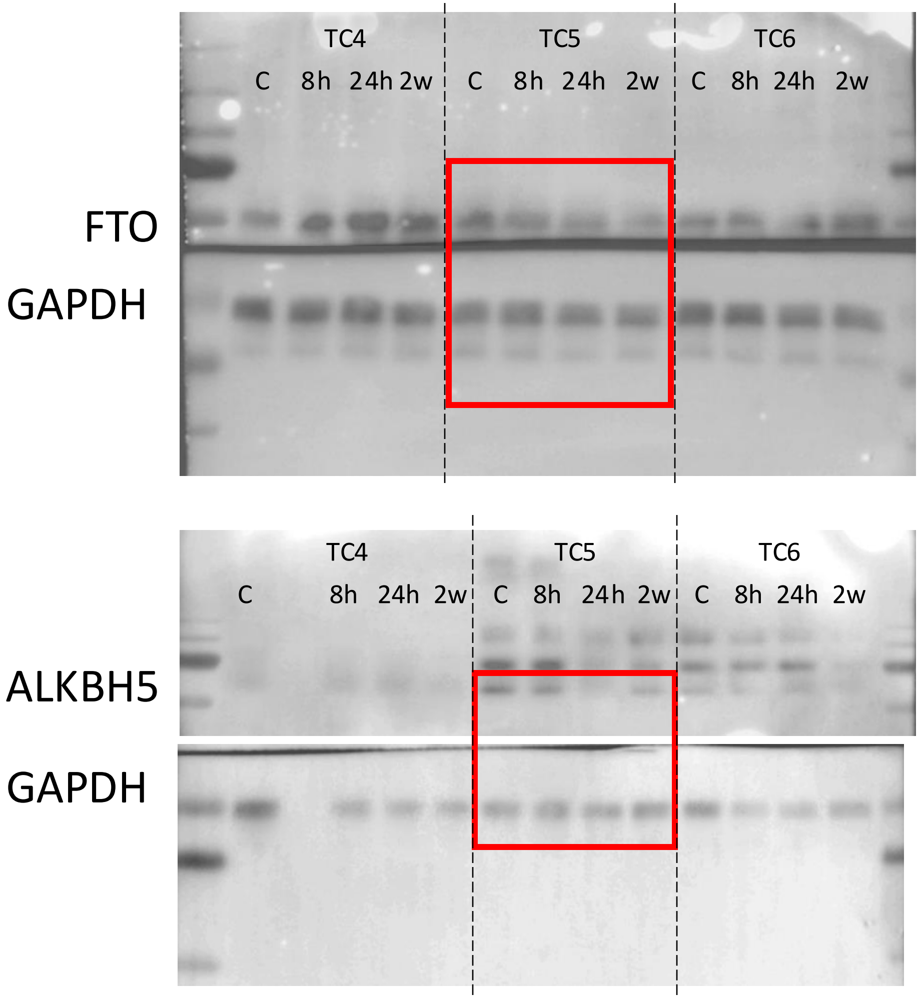

FTO
GAPDH
TC4
TC5
TC6
C 8h 24h 2w
C 8h 24h 2w
C 8h 24h 2w
ALKBH5
GAPDH
TC4
TC5
TC6
C 8h 24h 2w
C 8h 24h 2w
C 8h 24h 2w

# Figure 2

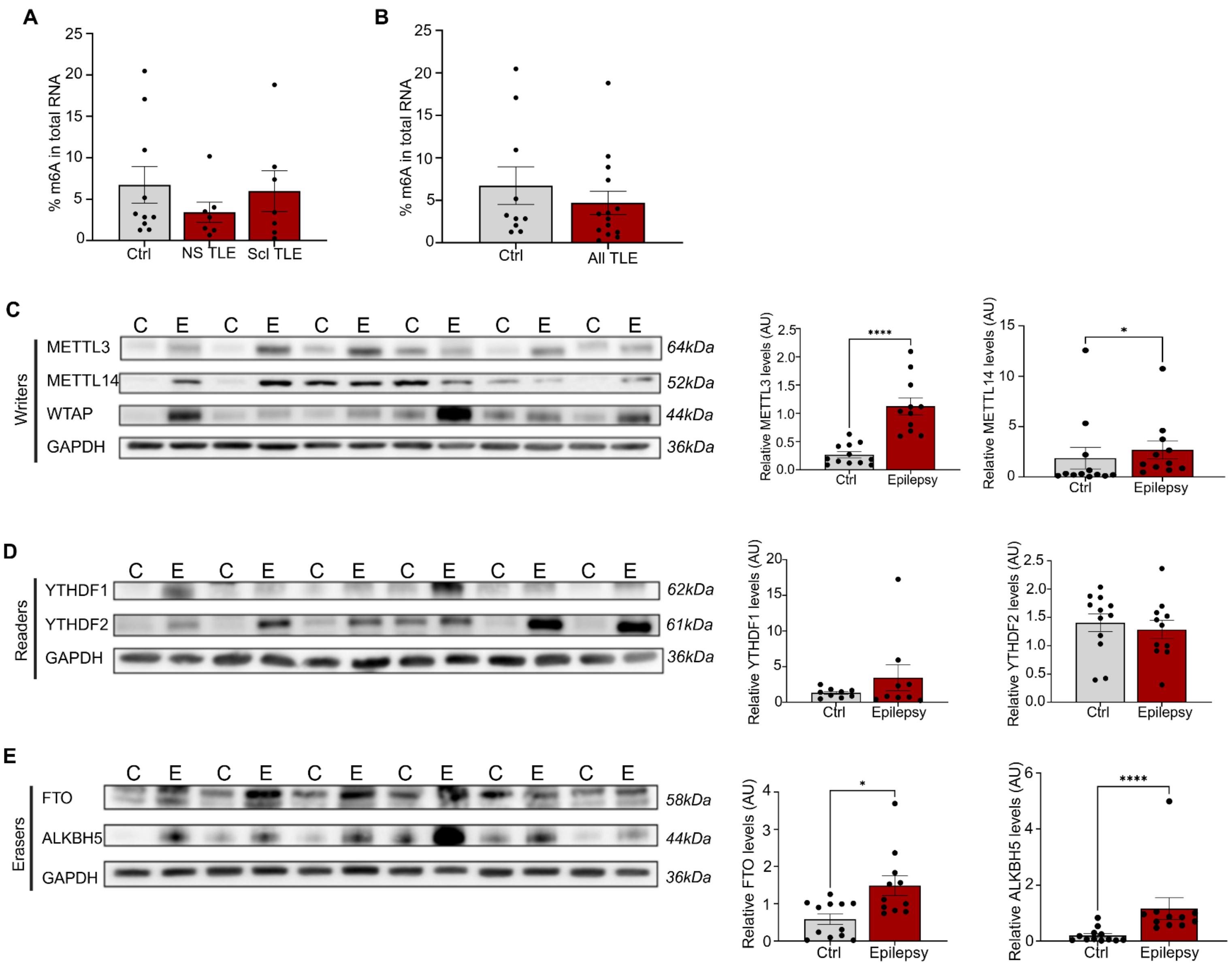

A
% m6A in total RNA
25
20
15
10
5
0
Ctrl
NS TLE
Scl TLE
B
% m6A in total RNA
Ctrl
All TLE
C
C E C E C E C E C E C E
Writers
METTL3 64kDa
METTL14 52kDa
WTAP 44kDa
GAPDH 36kDa
Relative METTL3 levels (AU)
Ctrl
Epilepsy
****
Relative METTL14 levels (AU)
*
D
Readers
YTHDF1 62kDa
YTHDF2 61kDa
GAPDH 36kDa
Relative YTHDF1 levels (AU)
Relative YTHDF2 levels (AU)
E
Erasers
FTO 58kDa
ALKBH5 44kDa
GAPDH 36kDa
Relative FTO levels (AU)
*
Relative ALKBH5 levels (AU)
****

**Figure 2C**

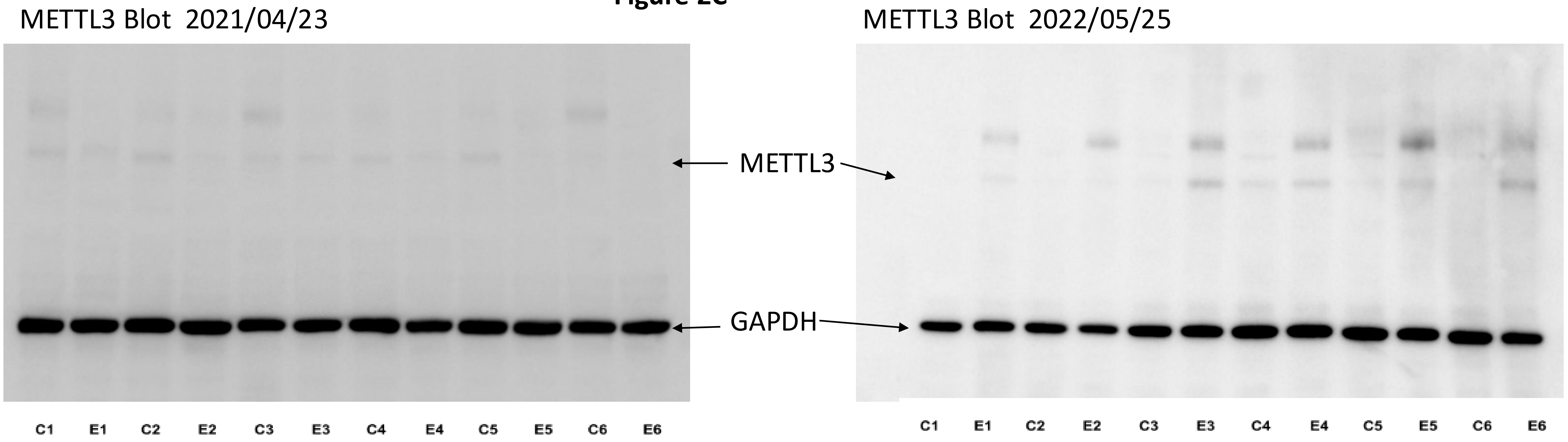
METTL3 Blot 2021/04/23
METTL3 Blot 2022/05/25
METTL3
GAPDH
C1 E1 C2 E2 C3 E3 C4 E4 C5 E5 C6 E6
C1 E1 C2 E2 C3 E3 C4 E4 C5 E5 C6 E6

**Figure 2C**

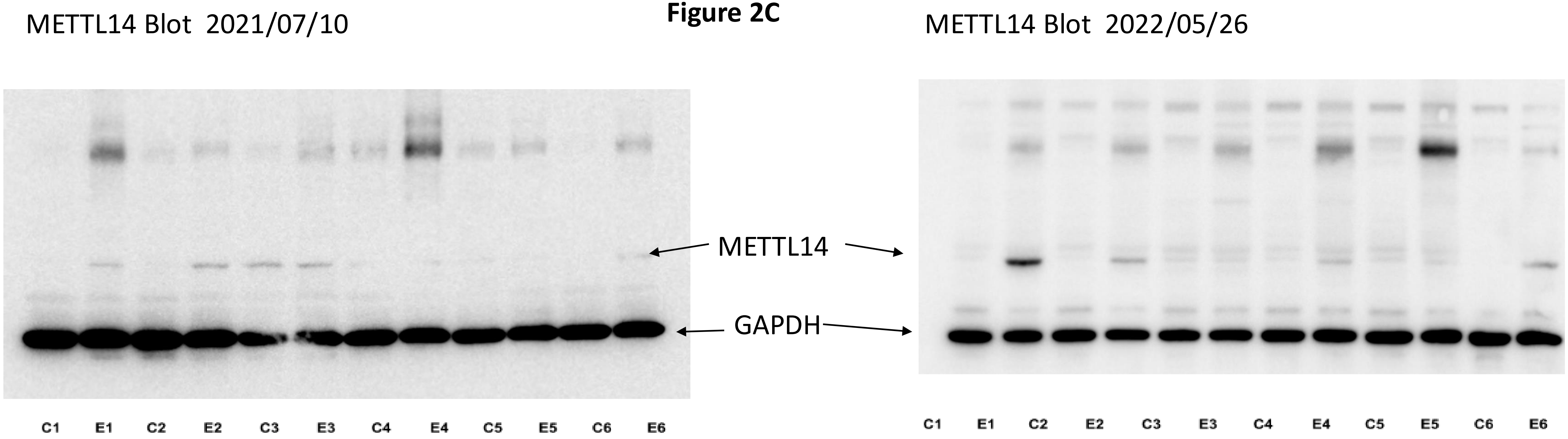
METTL14 Blot 2021/07/10
METTL14 Blot 2022/05/26
METTL14
GAPDH
C1 E1 C2 E2 C3 E3 C4 E4 C5 E5 C6 E6
C1 E1 C2 E2 C3 E3 C4 E4 C5 E5 C6 E6

**Figure 2C**

WTAP Blot 2021/08/19

WTAP Blot 2022/06/29

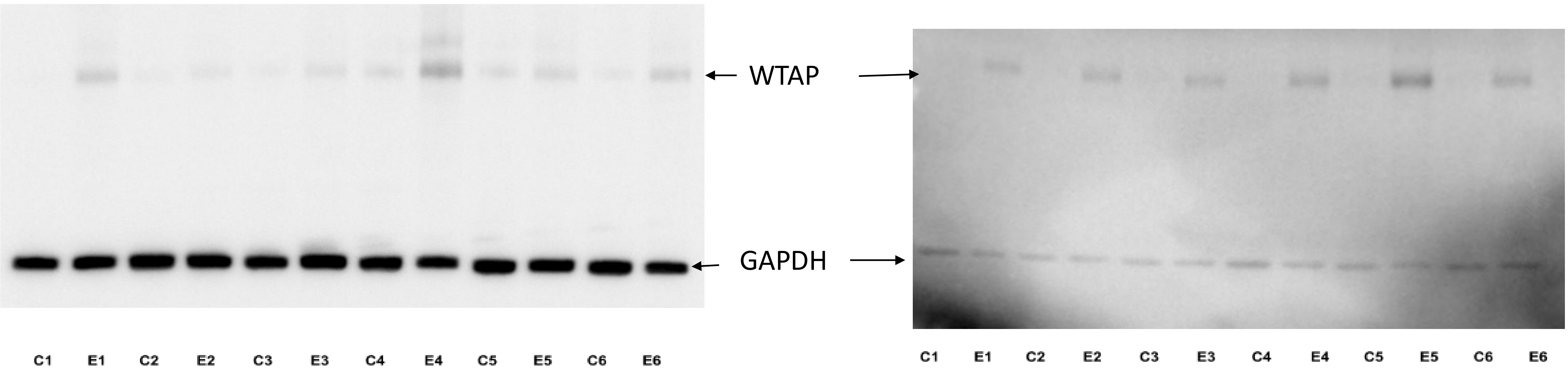

**Figure 2D**

YTHDF1* Blot 2021/07/07

YTHDF1 Blot 2022/04/13

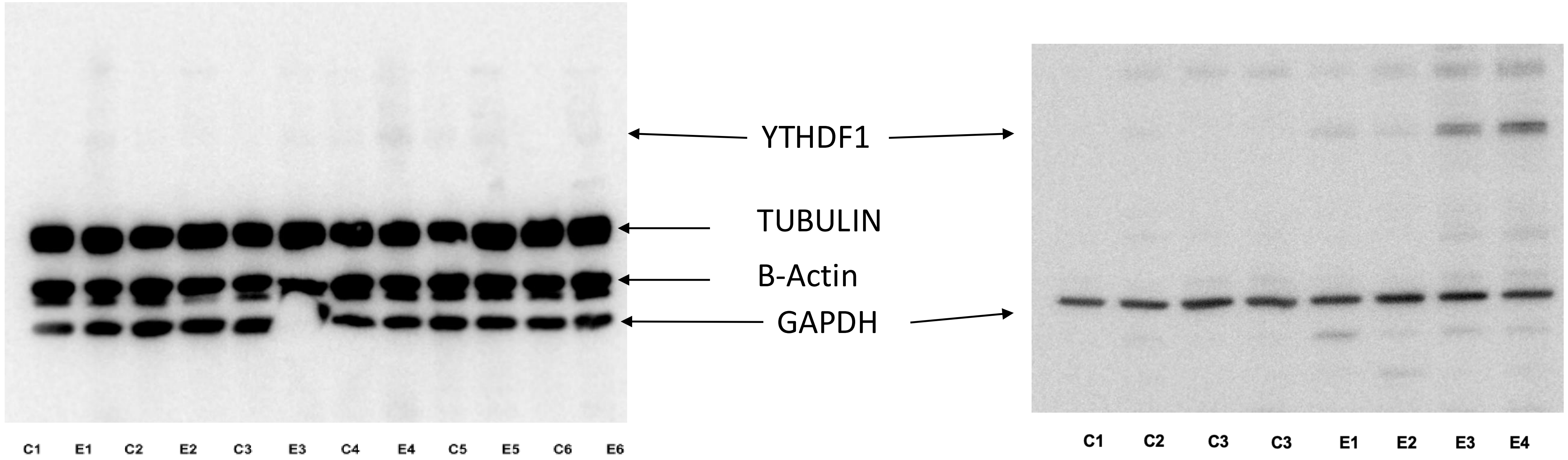


*Normalized with Tubulin as a bubble in the gel made GAPDH and B-actin normalization impossible.

YTHDF2 Blot 2021/07/29

**Figure 2D**

YTHDF2 Blot 2022/06/30

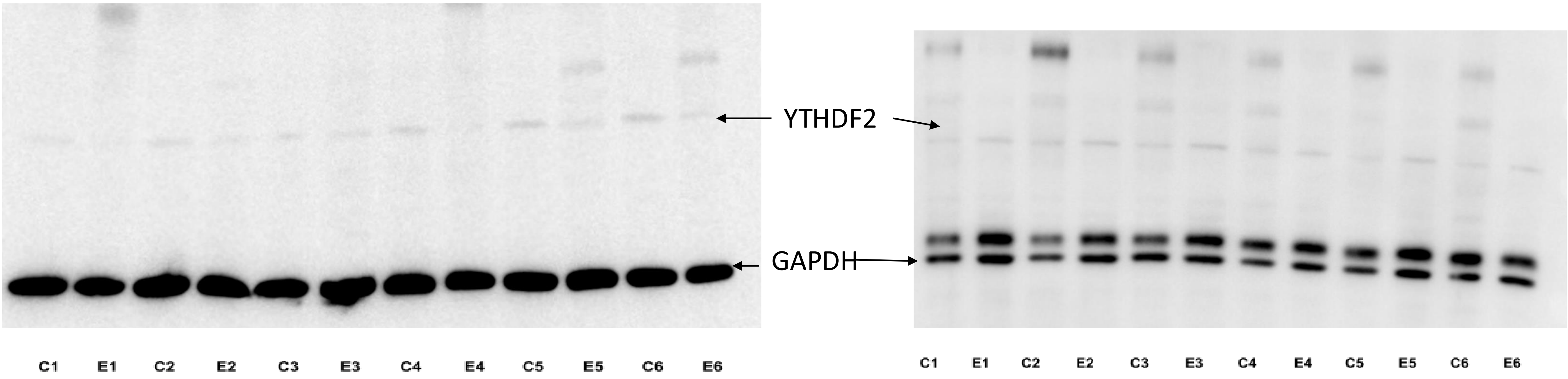

**Figure 2E**

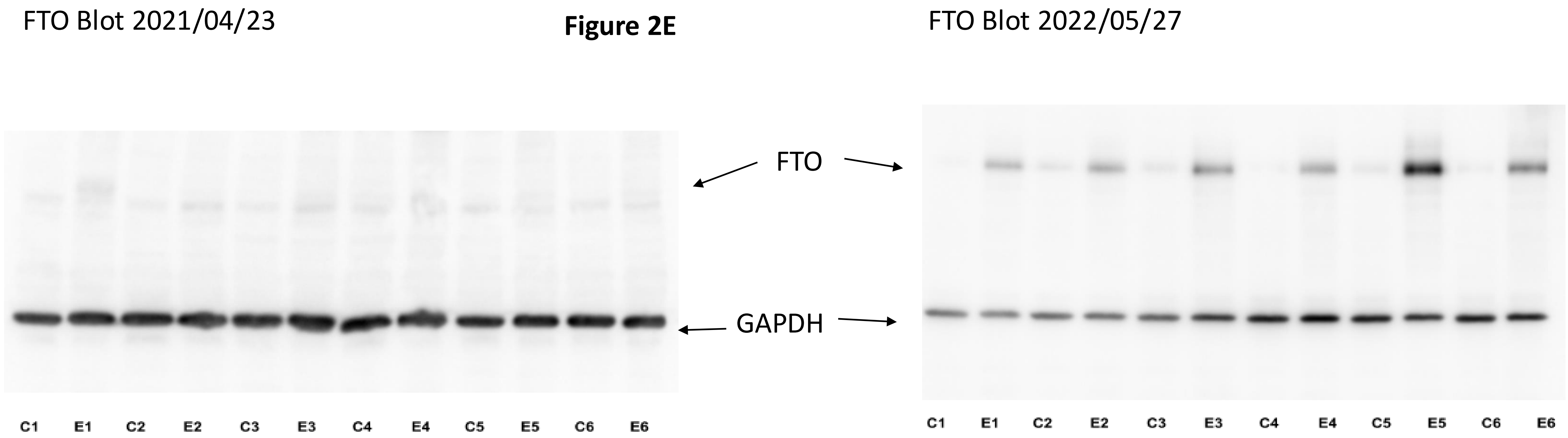
FTO Blot 2021/04/23
FTO Blot 2022/05/27
FTO
GAPDH
C1 E1 C2 E2 C3 E3 C4 E4 C5 E5 C6 E6
C1 E1 C2 E2 C3 E3 C4 E4 C5 E5 C6 E6

**Figure 2E**

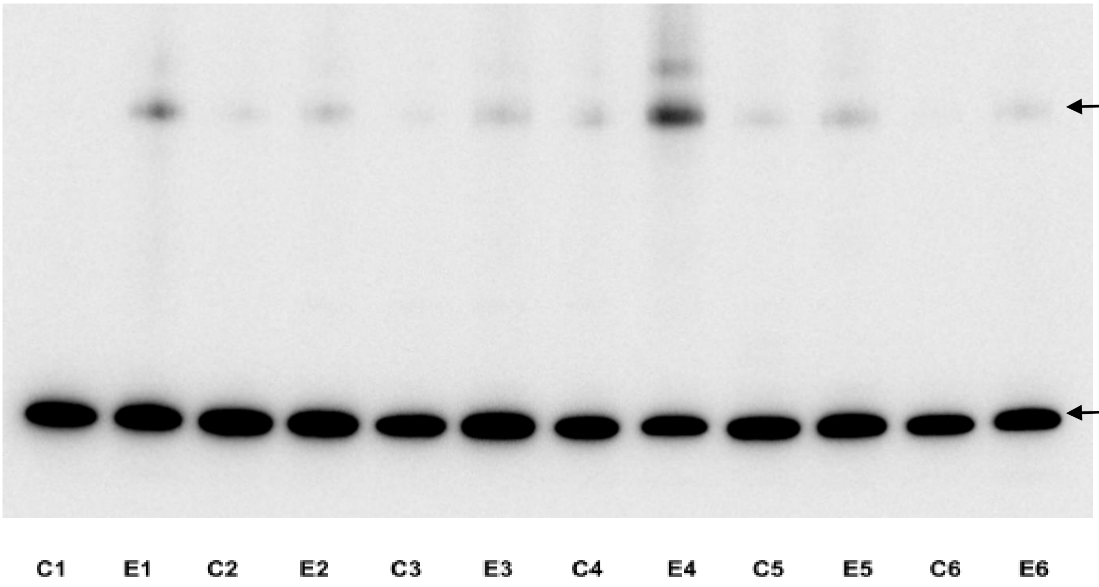
ALKBH5 Blot 2021/08/19
ALKBH5
GAPDH
C1 E1 C2 E2 C3 E3 C4 E4 C5 E5 C6 E6

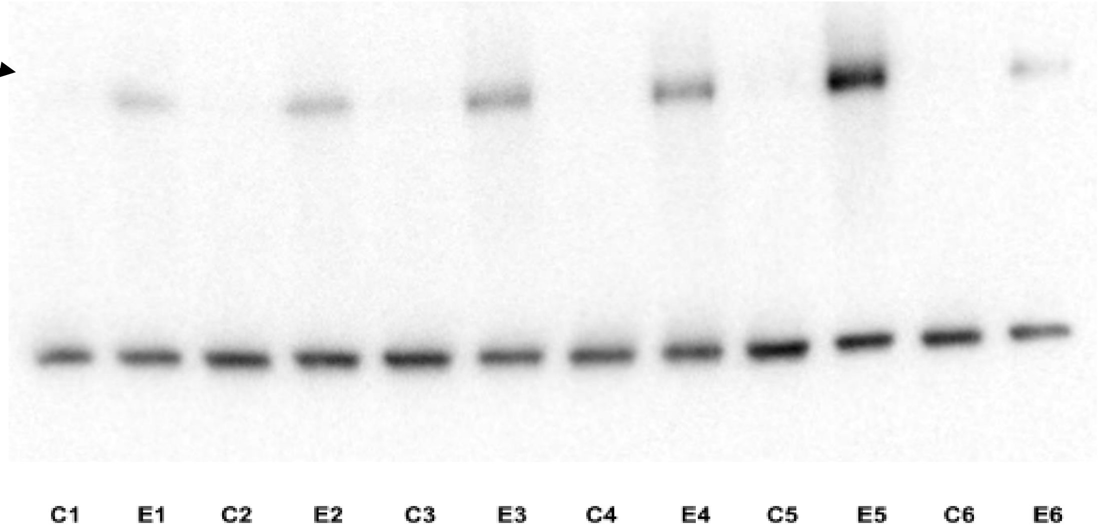
ALKBH5 Blot 2022/05/30
C1 E1 C2 E2 C3 E3 C4 E4 C5 E5 C6 E6

# Figure 8F

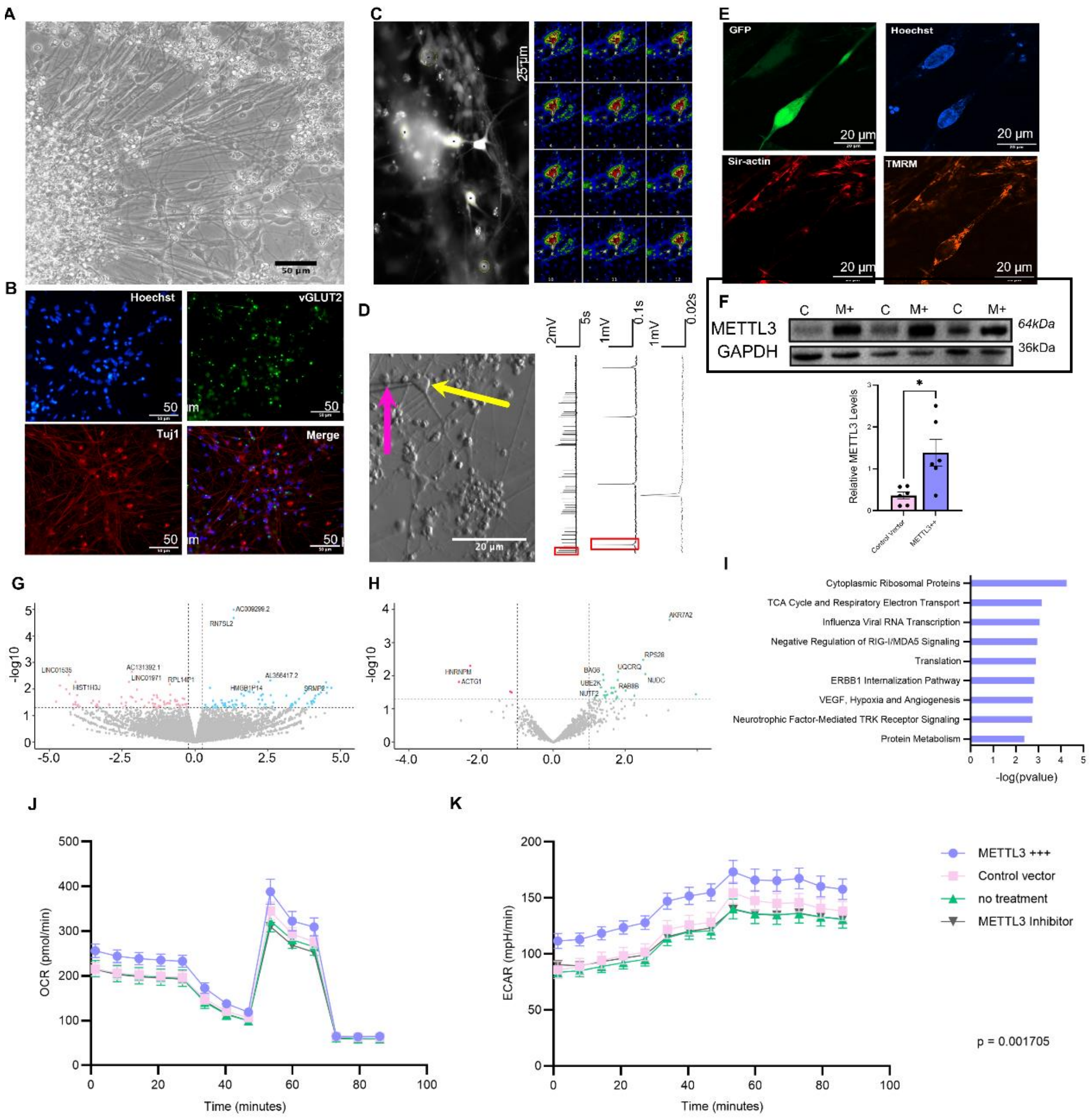


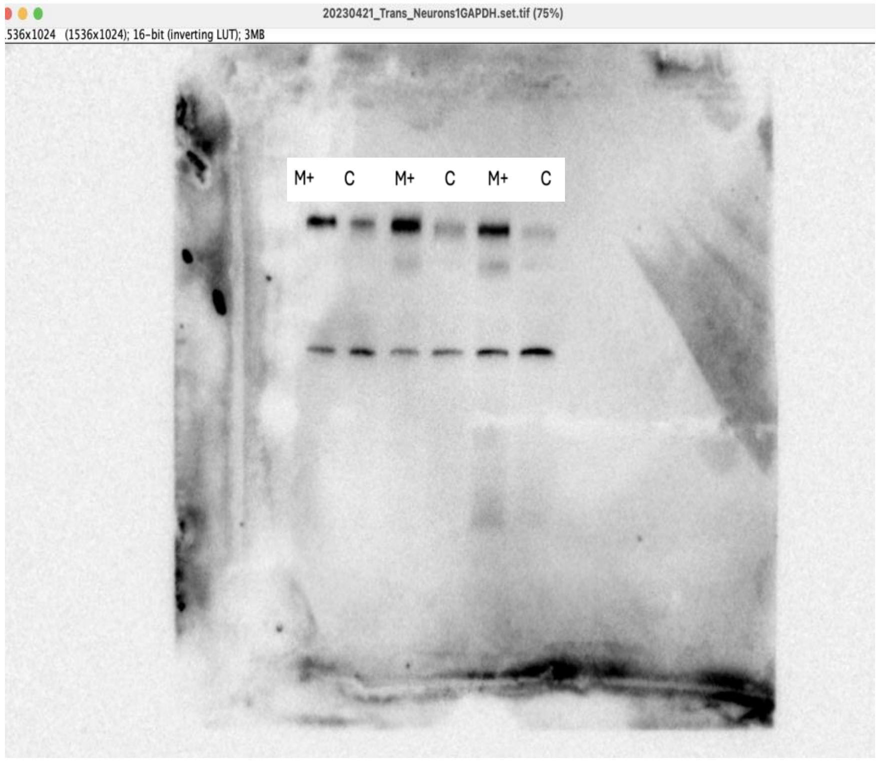


METTL3+

GAPDH

Image Flipped in paper to show control sample 1st

# Supplementary Figure 7A

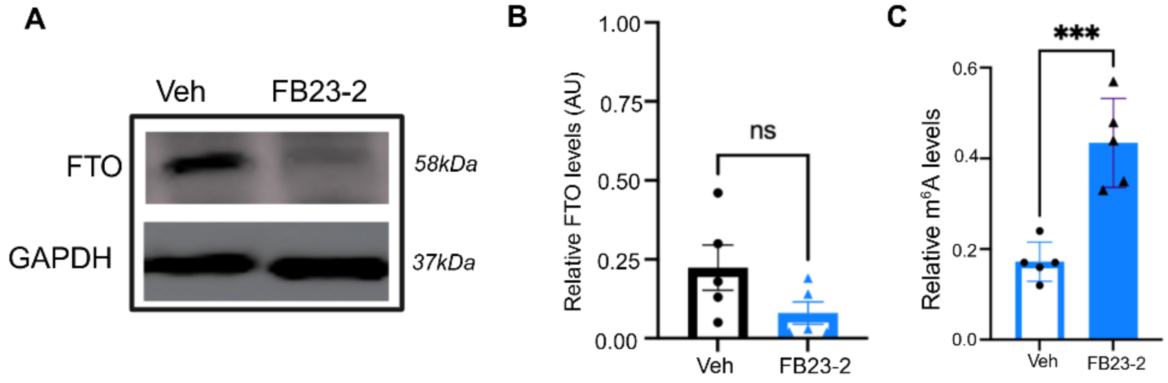


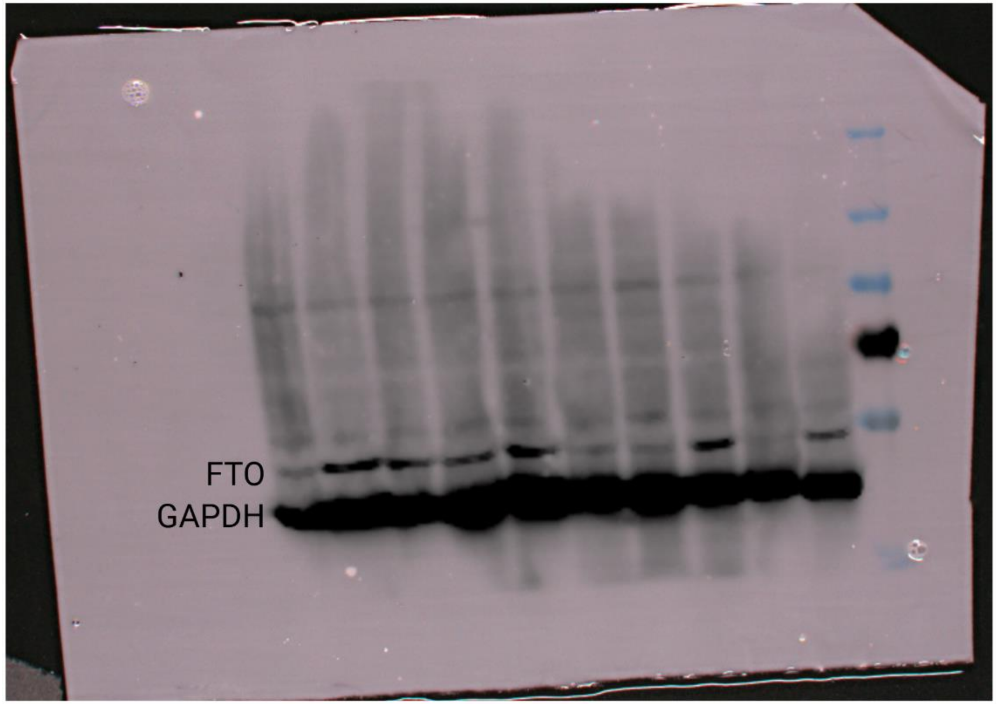

Supplement: Unedited blot and gel images [file jciinsight-10-188612-s068.pdf]
